# An insulator blocks access to enhancers by an illegitimate promoter, preventing repression by transcriptional interference

**DOI:** 10.1371/journal.pgen.1009536

**Published:** 2021-04-26

**Authors:** Miki Fujioka, Anastasiya Nezdyur, James B. Jaynes

**Affiliations:** Thomas Jefferson University, Philadelphia, Pennsylvania, United States of America; The University of North Carolina at Chapel Hill, UNITED STATES

## Abstract

Several distinct activities and functions have been described for chromatin insulators, which separate genes along chromosomes into functional units. Here, we describe a novel mechanism of functional separation whereby an insulator prevents gene repression. When the *homie* insulator is deleted from the end of a Drosophila *even skipped* (*eve*) locus, a flanking P-element promoter is activated in a partial *eve* pattern, causing expression driven by enhancers in the 3’ region to be repressed. The mechanism involves transcriptional read-through from the flanking promoter. This conclusion is based on the following. Read-through driven by a heterologous enhancer is sufficient to repress, even when *homie* is in place. Furthermore, when the flanking promoter is turned around, repression is minimal. Transcriptional read-through that does not produce anti-sense RNA can still repress expression, ruling out RNAi as the mechanism in this case. Thus, transcriptional interference, caused by enhancer capture and read-through when the insulator is removed, represses *eve* promoter-driven expression. We also show that enhancer-promoter specificity and processivity of transcription can have decisive effects on the consequences of insulator removal. First, a core *heat shock 70* promoter that is not activated well by *eve* enhancers did not cause read-through sufficient to repress the *eve* promoter. Second, these transcripts are less processive than those initiated at the P-promoter, measured by how far they extend through the *eve* locus, and so are less disruptive. These results highlight the importance of considering transcriptional read-through when assessing the effects of insulators on gene expression.

## Author summary

Several distinct activities and functions have been described for chromatin insulators, which are regulatory DNA elements that separate genes along chromosomes into functional units. Here, we describe how insulators can prevent repression of one gene by preventing inappropriate transcription of another gene, without blocking read-through of transcription *per se*. When the insulator *homie* is deleted from the end of a transgenic *eve* locus, a flanking transposable element promoter is activated by *eve* enhancers, causing repression of the *eve* promoter. The mechanism involves transcriptional read-through from the flanking promoter, which disrupts normal *eve* enhancer-promoter activities. When the flanking promoter is turned around, repression of *eve* is minimal. Thus, transcriptional interference, caused by enhancer capture and read-through when the insulator is removed, represses the *eve* promoter. These results show a novel role for transcriptional read-through in the effects of insulators on gene expression.

## Introduction

Recent studies have shown that insulators are multi-functional elements, performing several important tasks [1]. They block both enhancer-promoter (E-P) interactions [2–7] and the spreading of repressive histone modifications along the chromatin fiber [8–12]. At the same time, they form relatively stable interactions with other insulators, sometimes nearby and sometimes at great distances, thus contributing to chromosome organization [7,13–20]. When distant regions are brought together, novel E-P interactions can occur that contribute significantly to patterns of gene expression.

In Drosophila, insulators (a.k.a. boundaries) that separate adjacent *cis*-regulatory domains in the *bithorax* complex (BX-C) have been well studied [21–25]. For example, the *Fab-7* boundary separates the *iab-6* and *iab-7 cis*-regulatory elements of the *Abdominal-B* (*Abd-B*) gene that control phenotypes of parasegments (PSs) 11 and 12, respectively. Mutations that remove *Fab-7* cause transformation of PS11 to PS12, explained by ectopic activation of *iab-7* in PS11 due to a loss of enhancer blocking activity in early embryos. Later studies found that *Fab-7* is also required for insulating the repressive effects of a Polycomb Response Element (PRE) that prevents inappropriate activation of *Abd-B* in specific tissues [26,27]. Genome-wide analysis found these insulators to be enriched in insulator binding proteins [28].

Insulators are also involved in defining TADs (topologically associating domains) [1,29,30]. A recent study showed that removing the Fub boundary from the BX-C caused the fusion of two adjacent TADs [31], resulting in ectopic expression of *abdominal-A* (*abd-A*) in segment A1 [32,33]. Similarly, in a mammalian Hox complex, CTCF was shown to be required for insulating adjacent chromatin domains from each other [34,35]. While the majority of TADs in mammals are flanked by CTCF binding sites [36,37], CTCF is only one of a group of proteins that contribute to insulator function in Drosophila, and not all Drosophila insulators have CTCF binding sites [38]. Another difference between insulators in mammals and Drosophila is that mammalian TADs often contain several transcription units [39], while most transcription units in Drosophila, along with their regulatory DNA, constitute a separate TAD [40]. Despite these distinctions, mammalian insulators probably perform the same range of functions as those in Drosophila.

Roles for nuclear RNAs in regulating chromatin structure and gene expression have been uncovered in recent years [41–43]. Transcription itself, proceeding through a gene, may result in transcriptional interference. Such read-through may disrupt either enhancer or core promoter function, or disrupt their ability to interact over long distances. Mechanistically, read-through may disrupt transcription factor binding or function [44]. Read-through transcription can underlie disruptions in development. For example, insertion into the BX-C of promoters contained within either the 5’ P-element end [45] or the *scs* insulator [46] have been shown to produce read-through transcripts driven by nearby enhancers that cause disruptions of gene expression, and consequent developmental defects. Conversely, a 92 kb non-coding RNA, which is normally initiated within the *iab-8* regulatory domain of the BX-C and reads through several insulators, is required for repression of *abd-A* in parts of the nervous system, so that disrupting it causes defects [47].

Segmentation is very sensitive to the levels of *eve* expression, so that even minor changes cause developmental defects, making manipulations of the endogenous locus problematic [48,49]. In order to circumvent this limitation, we previously created a pseudo-locus transgene [12], which contains the entire *eve* locus and the 5’ end of the 3’-neighboring gene, *TER94*. In this pseudo-locus, the *eve* protein coding region is replaced by that of *lacZ*, while that of *TER94* (an essential, ubiquitously expressed gene) [50–52] is replaced by EGFP. Using the φC31 integrase system [53], this pseudo-locus was inserted at several attP docking sites, which had been introduced into the genome by P-element transformation [12]. Removing the 3’ *eve* insulator *homie* from the pseudo-locus reduced *eve-lacZ* activity, particularly that of enhancers located 3’ of the transcription unit [54,55]. This was seen as reduced expression of early stripes 1, 4, 5, and 6, as well as reduction of later-stage, tissue-specific expression [12]. Early stripes 2, 3, and 7 are driven by enhancers upstream of the start site [56–59], and they were repressed less, giving rise to a clear imbalance in early stripe expression. This kind of early stripe imbalance, when it occurs at a functional *eve* locus, causes severe, lethal disruptions in segmentation, illustrating the value of using the pseudo-locus to study *eve* regulation [48,49].

Here, we focus on the two insulators that flank the *eve* locus, *homie* and *nhomie* [7,12,19]. We show that the stripe imbalance seen at the pseudo-locus when an insulator is removed is caused by the capturing of *eve* enhancer activity by a flanking P-element promoter [60–63]. The absence of an insulator allows the flanking promoter to be activated by *eve* enhancers in a pattern that matches the pattern of repression of *eve-lacZ*. We further show that the repression is primarily attributable to transcriptional interference by read-through, with a minor contribution from promoter competition. The imbalance is not prevented by the insertion of multiple poly-adenylation (polyA) signals from the *α-tubulin* gene [64] and *SV40* [65], because it does not effectively stop transcriptional read-through. A commonly used core promoter from the *heat shock 70* (*hsp70*) locus [66] does not produce such processive transcripts, and so does not disrupt *eve-lacZ* expression. These studies add to our knowledge of the mechanisms underlying disruptions of gene expression seen when insulators are removed, or their functions compromised.

## Results

### Removal of an insulator causes disruption of *eve* promoter expression by a flanking activity

**Fig 1:**
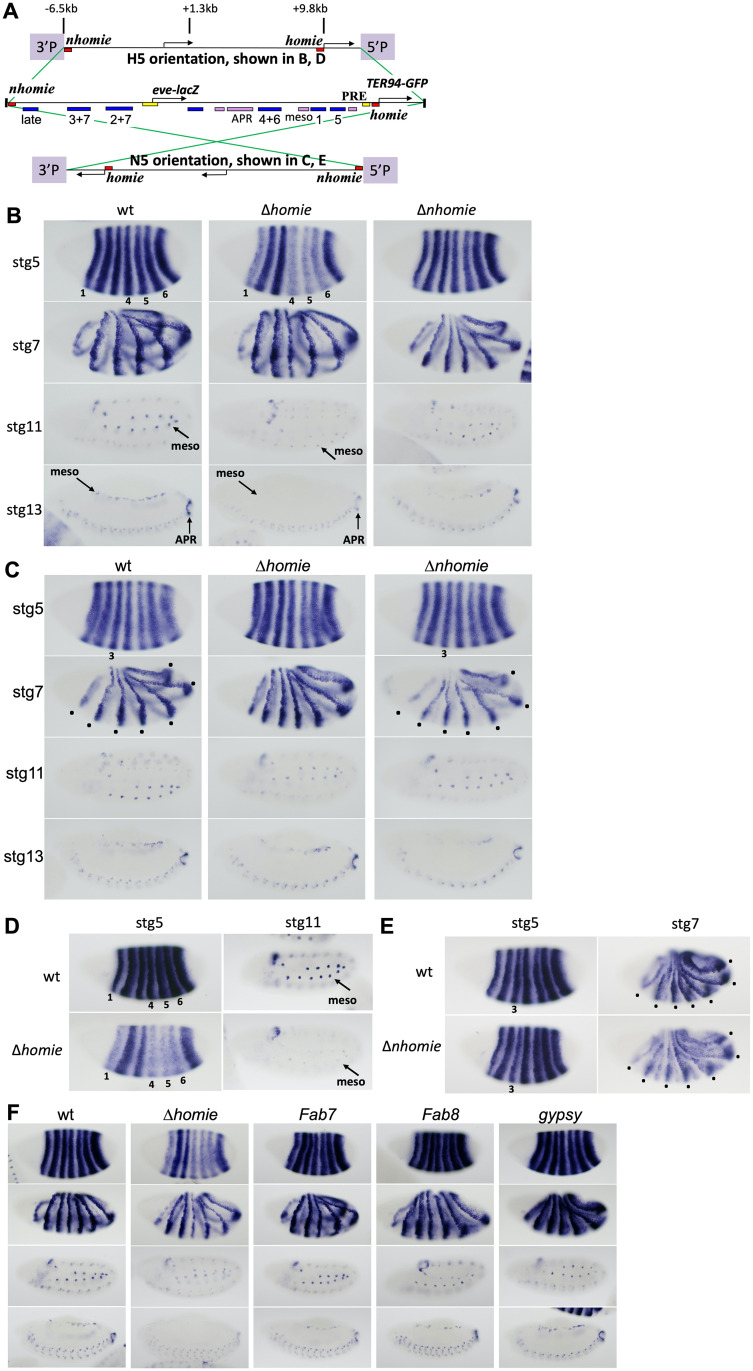
Effects on expression due to *eve* insulator removal and replacement. **(A)** Map of the *eve* pseudo-locus construct (inserted in either orientation, H5 or N5). The wild-type *eve* and *TER94* coding regions were replaced with *lacZ* and *EGFP*, respectively. Rectangles represent regulatory elements: red, *nhomie* and *homie* insulators; yellow, Polycomb response elements (PREs); blue, embryonic stripe enhancers labeled with either numbers for early stripes, or “late” for the 7 later stripes; pink, tissue specific enhancers for: APR, anal plate ring; meso, mesoderm (other known elements are either unlabeled or not included). 5’P and 3’P: 5’ and 3’ P-element ends, part of the *attP* site previously inserted at cytological location 74A2 or 23C4. Note that the 5’P end contains a P-element promoter, but the 3’P end does not. **(B)** Inserted in the H5 orientation at site 74A2. Rows: *lacZ* expression (visualized by RNA *in situ* hybridization) at embryonic stages 5, 7, 11, 13. Columns: wt: “wild-type” intact pseudo-locus construct; Δ*homie*: 500 bp *homie* was replaced with a similar length of phage λ DNA; Δ*nhomie*: 200 bp core *nhomie* was deleted. Affected tissues are labeled, including stripes number 1, 4, 5, and 6 at stg5, and mesoderm (meso) and anal plate ring (APR) at stage (stg) 11 and stg13. **(C)** Same as in A, but in the N5 orientation. Early stripe 3 at stg5 is marked (note the reduction in intensity of stripe 3 relative to flanking stripes in Δ*nhomie* vs. the same comparison in wt). The 7 late stripes at stg7 are marked with dots (note the reduction in intensity in Δ*nhomie* relative to wt). **(D)** In the H5 orientation at site 23C4. wt (top row) and Δ*homie* (bottom row) are shown at stg5 and stg11. The affected expression is marked with numbers and "meso". **(E)** In the N5 orientation at site 23C4. wt (top row) and Δ*nhomie* (bottom row) are shown at stg5 and stg7. Affected expression is marked with “3” and dots. **(F)** 500 bp *homie* was replaced with either a *Fab7*, *Fab8*, or *gypsy* insulator in the H5 orientation. *lacZ* expression is shown at stages 5, 7, 11, and 13.

For this study, we added *nhomie* [19] to the 5’ end of *eve-lacZ*, making the pseudo-locus identical to the endogenous *eve* locus including the 5’ end of *TER94*, except for the replacement of protein coding sequences (Fig 1A). Recombinase-mediated cassette exchange (RMCE) [53] was used for transgene insertion, which could occur in either orientation at each site, and both orientations were analyzed. The control construct faithfully reproduced the full *eve* expression pattern throughout embryogenesis, independent of the orientation of the insert (Fig 1B and 1C, “wt”). In one orientation of the pseudo-locus (the H5 orientation, *homie* is close to 5’P, Fig 1A and 1B), removing *homie* disrupted *eve-lacZ* expression [12], causing early stripes 1, 4, 5, and 6 to be reduced relative to stripes 2, 3, and 7, along with repression of all later tissue-specific expression (including in the mesoderm, "meso", and anal plate ring, "APR", Fig 1A and 1B). In the other orientation (N5, *nhomie* is close to 5’P, Fig 1A and 1C), the expression pattern without *homie* was indistinguishable from that of the wild-type locus, although there may be a tendency toward slightly weakened *eve-lacZ* expression overall, as well as occasional stripe imbalances (Fig 1C, “Δ*homie*”). However, when *nhomie* was removed in this N5 orientation, both early stripe 3 at stage 5 (Fig 1C, “Δ*nhomie*”, compare stripe 3 intensity to stripe 4 at “stg5”) and the later 7-stripe expression at stage 7 (driven by the enhancer labeled “late” in Fig 1A) was somewhat reduced (Fig 1C, “Δ*nhomie*”, black dots mark affected stripes at stage 7). In contrast, in the H5 orientation where removal of *homie* had a dramatic effect, removing *nhomie* had little effect (Fig 1B, Δ*nhomie*; here again, there may be slightly weakened *eve-lacZ* expression overall, as well as occasional stripe imbalances). We tested the same pseudo-locus transgenes inserted at other P-element-attP sites, and they showed the same tendencies, but with even stronger repressive effects when the insulator at the “5’P” end of the locus was removed (Fig 1D and 1E). Therefore, removing either *homie* or *nhomie* affects different sets of enhancers, and the effects strongly depend on the orientation of the transgenic *eve* locus, implicating a specific flanking activity in causing the disruptions.

Insulators are known to block both E-P interactions and the spreading of repressive chromatin from a nucleation site, such as a PRE [1,7,12]. Is the flanking activity that is responsible for *eve* pseudo-locus disruption either of these known insulator-blocked activities? As a first step to test this, several other insulators were used to replace *homie* in the pseudo-locus in the H5 orientation, and we asked whether they could prevent repression of *eve-lacZ*. All transgenes with either the *Fab-7*, *Fab-8*, or *gypsy* insulators expressed *eve-lacZ* similarly to wild-type (Fig 1F), showing that they all can protect the *eve* locus. Thus, this protective activity is a common feature of insulators.

### The flanking disruptive activity is attributable to a transposable element promoter

**Fig 2:**
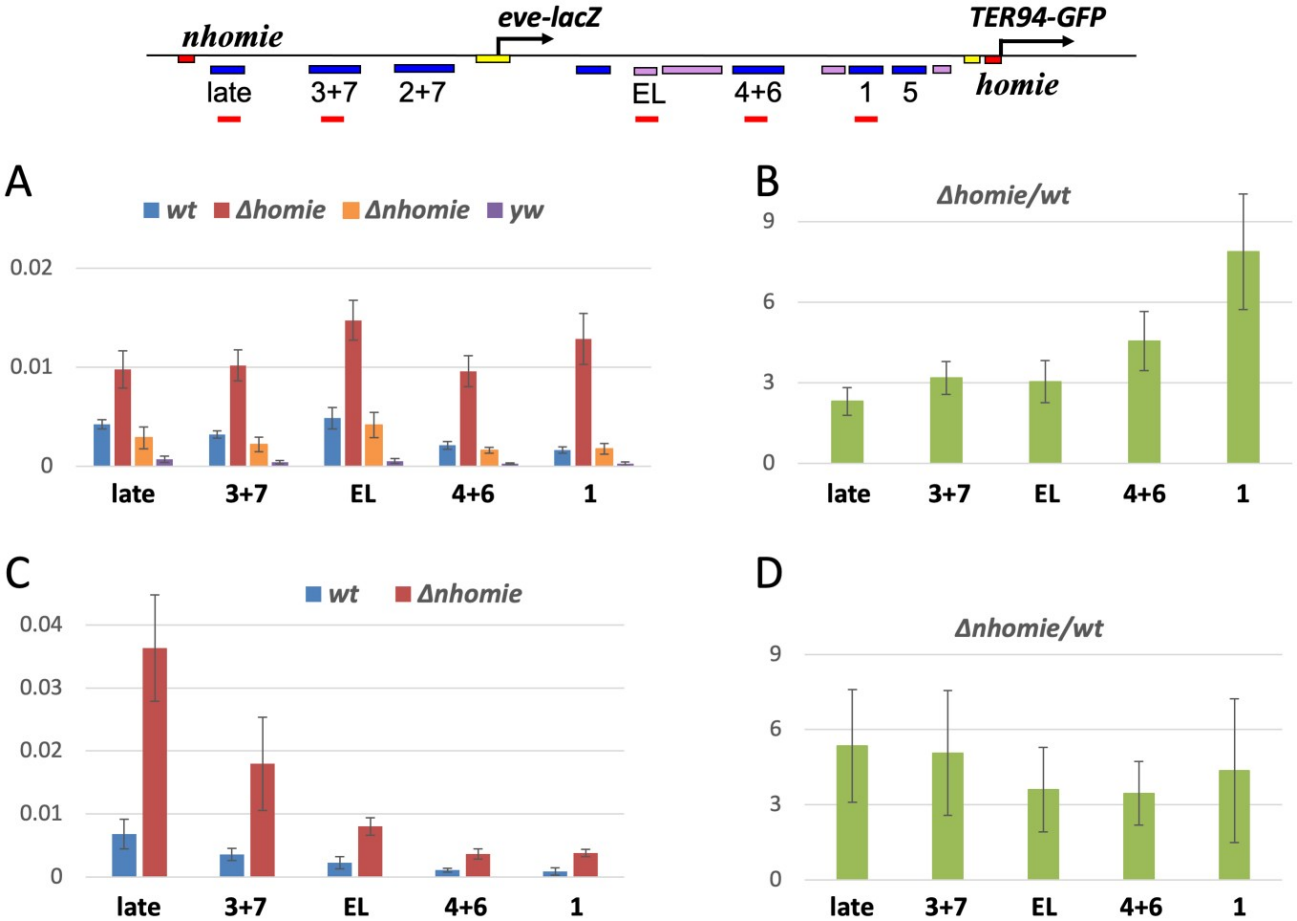
Non-coding RNA expression is increased by *eve* insulator removal. **Top map**: locations of amplification products quantified by qPCR are shown as red bars. **(A, B)** transgenes in the H5 orientation (see Fig 1A and 1B). **(A)** RT-qPCR quantification of total RNA (normalized to *RP49* RNA) from the indicated enhancer regions, in the wt, Δ*homie*, and Δ*nhomie* transgenic lines (shown in Fig 1B), and in a *yw* control (no transgene: signal comes only from endogenous *eve*). Averages with standard deviations of 3 biological samples are graphed. Note that transcript levels are increased throughout the locus in Δ*homie*, but not in Δ*nhomie*. **(B)** The ratios of average signals from Δ*homie* and wt in A are graphed (with standard deviations). **(C, D)** transgenes in the N5 orientation (see Fig 1A and 1C). **(C)** Similar to A, except that wt and Δ*nhomie* are at the insertion site shown in Fig 1C. **(D)** The ratios of average signals (with standard deviations) from Δ*nhomie* and wt in C are graphed.

What is causing repression of *eve-lacZ* in the absence of an intervening insulator? In all cases where removing *homie* or *nhomie* had a strong effect, the repressed enhancers were located toward the end of the locus flanked by the 5’P-element end (a.k.a. P-element border), which contains a P-element promoter [60–63]. Therefore, we hypothesized that the P-element promoter was driving read-through transcription, and that this was involved in repressing *eve-lacZ* enhancer activity. To begin to test this, we quantified transcripts in the *eve* regulatory regions using RT-qPCR. First, we found that transcripts were barely detectable throughout the endogenous *eve* locus (using *yw* flies not containing the pseudo-locus, Fig 2A, “*yw*”). With the wild-type pseudo-locus, slightly higher transcript levels were seen (Fig 2A, “wt” vs. “*yw*”). However, without *homie* (Fig 2A, “Δ*homie*”), transcript levels were increased. Consistent with our hypothesis, deletion of *nhomie* in the H5 orientation showed similar levels of transcripts to wild-type (Fig 2A, "Δ*nhomie*"). This is potentially attributable to a blocking activity of insulators, since *nhomie* in the H5 orientation is not between the *eve* enhancers and P-element promoter (see Fig 1A map). In contrast, in the N5 orientation, deletion of *nhomie* caused increased transcript levels, while the transcript levels remained low in wild-type (Fig 2C, “Δ*nhomie*”, compare to "wt"). The ratio of Δ*homie* or Δ*nhomie* transcripts to wild-type showed a maximum near the 5’P-element end, and tended to decrease throughout the locus (Fig 2B and 2D). Similar effects were seen at another transgene landing site (S1 Fig). These data suggest that the absence of an insulator causes transcription through the *eve* enhancers, which is strongest through those enhancers that are most disrupted. This, in turn, may suggest that transcription through *eve* enhancers represses *eve-lacZ* expression.

**Fig 3:**
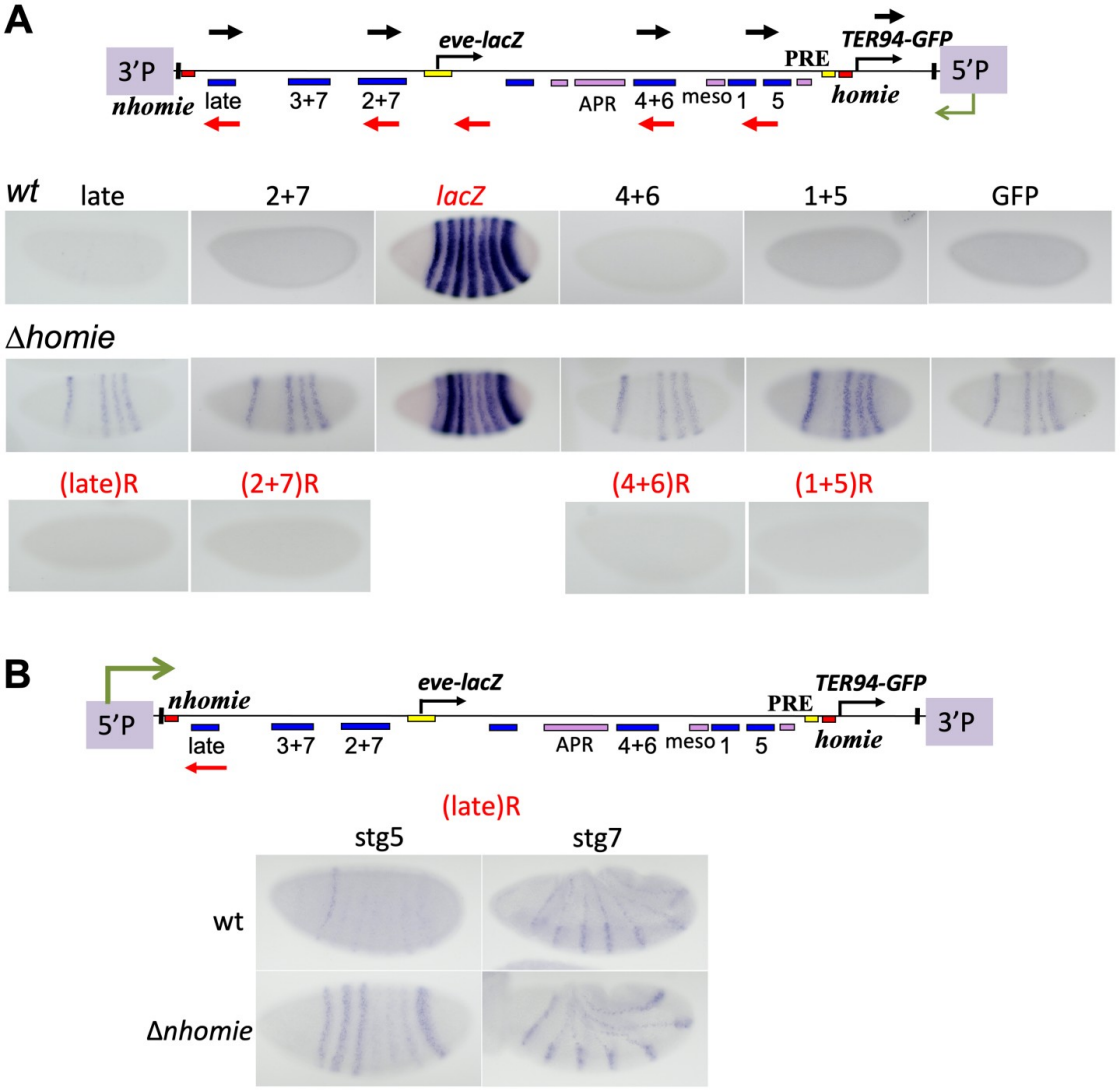
Patterns of non-coding RNA expression and *eve* promoter repression caused by insulator removal. RNA *in situ* hybridization to embryos carrying either the wt pseudo-locus, or Δ*homie* (in A), or Δ*nhomie* (in B). Probes are indicated with either black arrows, recognizing transcripts from downstream, or red arrows, recognizing transcripts from upstream. Images are labeled with either black lettering corresponding to black arrows, or red lettering (with ‘R’) corresponding to red arrows. **Maps**: Transgene constructs, showing location of the 5’ P-element end, where read-through transcripts are initiated. **(A)** Stage 5 embryos carrying the H5 orientation transgene at cytological location 74A2 (as in Fig 1B). **Top row**: wt embryos stained using the probes that recognize transcripts from downstream, where the P-element promoter is located ("late", "2+7", "4+6", "1+5", GFP). **Middle row**: Δ*homie* embryos, also stained using probes that recognize transcripts from downstream. **Bottom row**: Δ*homie* embryos stained using probes that recognize transcripts from upstream ("(late)R", "(2+7)R", "(4+6)R", "(1+5)R"). **(B)** Stage 5 and 7 embryos carrying either the wt (top row) or Δ*nhomie* transgene (bottom row) in the N5 orientation, at site 74A2 (used in Fig 1C). Transcripts coming from upstream, where the P-element promoter is located, are detected using probe (late)R (red arrow).

What is activating the P-element promoter? The *TER94* portion of the pseudo-locus contains ubiquitously active enhancers [12]. In a previous study, we showed that *TER94-GFP* is activated by the *eve* enhancers when *homie* is removed [12]. It is possible that the P-element promoter is activated by *TER94* enhancers, and/or by enhancers flanking the landing site. Therefore, we analyzed embryos using *in situ* hybridization to see the expression pattern of transcripts reading through *eve* enhancers. The *eve* regulatory regions were surveyed using *in situ* RNA probes that detect transcripts coming from the 5’P end. In the wild-type pseudo-locus in the H5 orientation, there was no detectable expression pattern in most of the enhancer regions (Fig 3A, “wt”, probes 2+7, 4+6, 1+5, GFP, black arrows above the map). The one exception is very faint expression at the late stripe enhancer, in the pattern of stripes 1, 2, 3, and 7 (Fig 3A, “wt”, “late” probe). In contrast, when *homie* was deleted, early stripe 1, 4, 5, and 6 expression was clearly observed throughout the *eve* locus (Fig 3A, “Δ*homie*”, probes late, 2+7, 4+6, 1+5, GFP), along with faint stripes 2, 3, and 7. Importantly, this expression pattern correlates with the pattern of repression of *eve-lacZ* (Fig 3A, *lacZ* probe, “Δ*homie*” vs. “wt”). In order to test whether this non-coding RNA expression is unidirectional, consistent with its coming from the P-element promoter, we used probes that recognize the opposite strand. With these probes, no detectable expression pattern was observed in any of the enhancer regions (Fig 3A, Δ*homie*, all probes tested).

In the N5 orientation, weak expression in the pattern of both early stripes and the 7 late stripes was observed downstream of the P-promoter, even in the presence of *nhomie* (Fig 3B, “wt”, probe (late)R). When *nhomie* was deleted, considerably stronger expression of early stripes 2, 3, 7, as well as somewhat stronger expression of stripe 1 and the 7 late stripes, was observed (Fig 3B, “Δ*nhomie*”, probe (late)R). Weak stripe 1 expression has been shown to be driven by sequences upstream of the *eve* start site [58], in addition to the stronger expression driven by the stripe 1 enhancer located near the 3’ end of the locus [55]. Therefore, all of these aspects of *eve* expression are driven by enhancers upstream of the *eve* promoter. This expression pattern can be seen throughout the locus, suggesting that the transcripts detected are continuous, long non-coding RNAs (S2 Fig, Δ*nhomie*, probes (late)R, (2+7)R, (4+6)R, (1+5)R). We did not observe any expression pattern with probes that recognize the opposite strand (S2 Fig, Δ*nhomie*, probes 2+7, 4+6, 1+5), although there are faint stripes with “late” probe (S2 Fig, Δ*nhomie*), similar to the ones seen in the H5 orientation (described above).

In order to determine whether the transcripts we detected were continuous, and not, for example, short eRNAs initiated within each enhancer, we further analyzed transcripts of the lines in the H5 orientation (used in Fig 1B, "wt" and "Δ*homie*") using RT-qPCR. We produced cDNA using specific primers to late or EL enhancer regions, which anneal to transcripts coming from the direction of the P-element promoter (S3 Fig, green arrows above the map). If the transcripts were continuous, using these cDNAs as template, we would detect qPCR amplification products at downstream enhancer regions. Indeed, we observed high qPCR signals at downstream enhancer sequences in the Δ*homie* line. The cDNA primed from the “late” enhancer is continuous to the 3+7 enhancer region, and the cDNA from the “EL” region is continuous to the 4+6 and 1+5 enhancer regions, but not to a region beyond the P-element promoter (“wD5”, S3A Fig), consistent with the expected initiation of transcripts at the P-promoter. In summary, the expression patterns show that the P-element promoter is driven by *eve* enhancers, which is permitted when the intervening insulator is removed, with expression being stronger for those enhancers that are closer to it. This then causes read-through that represses *eve-lacZ* expression. Thus, the relevant insulator mechanism preventing repression of the *eve* promoter may be primarily the enhancer blocking function.

In order to confirm that the 5’P-element end is the source of the transcriptional read-through, we used a MiMIC line [67], which does not carry a flanking P-element promoter. Without *nhomie*, the *eve-lacZ* pattern was similar to that of the wild-type pseudo-locus shown in Fig 1B, in either orientation (S4A Fig, Δ*nhomie*), although we detected some stripe imbalances when both *homie* and *nhomie* were deleted (S4A Fig, *Δnhomie*, *Δhomie*). However, these imbalances were more variable and less severe than those seen in Fig 1B and 1D, and were similar to those seen frequently when *eve* rescue constructs lacking the insulators were inserted in random locations throughout the genome [48,55]. Therefore, P-element promoter activity driven by the *eve* enhancers is the main cause of the repression of *eve-lacZ* expression in the absence of an intervening insulator.

### Transcriptional read-through is essential for the disruptive effect of the flanking promoter

**Fig 4:**
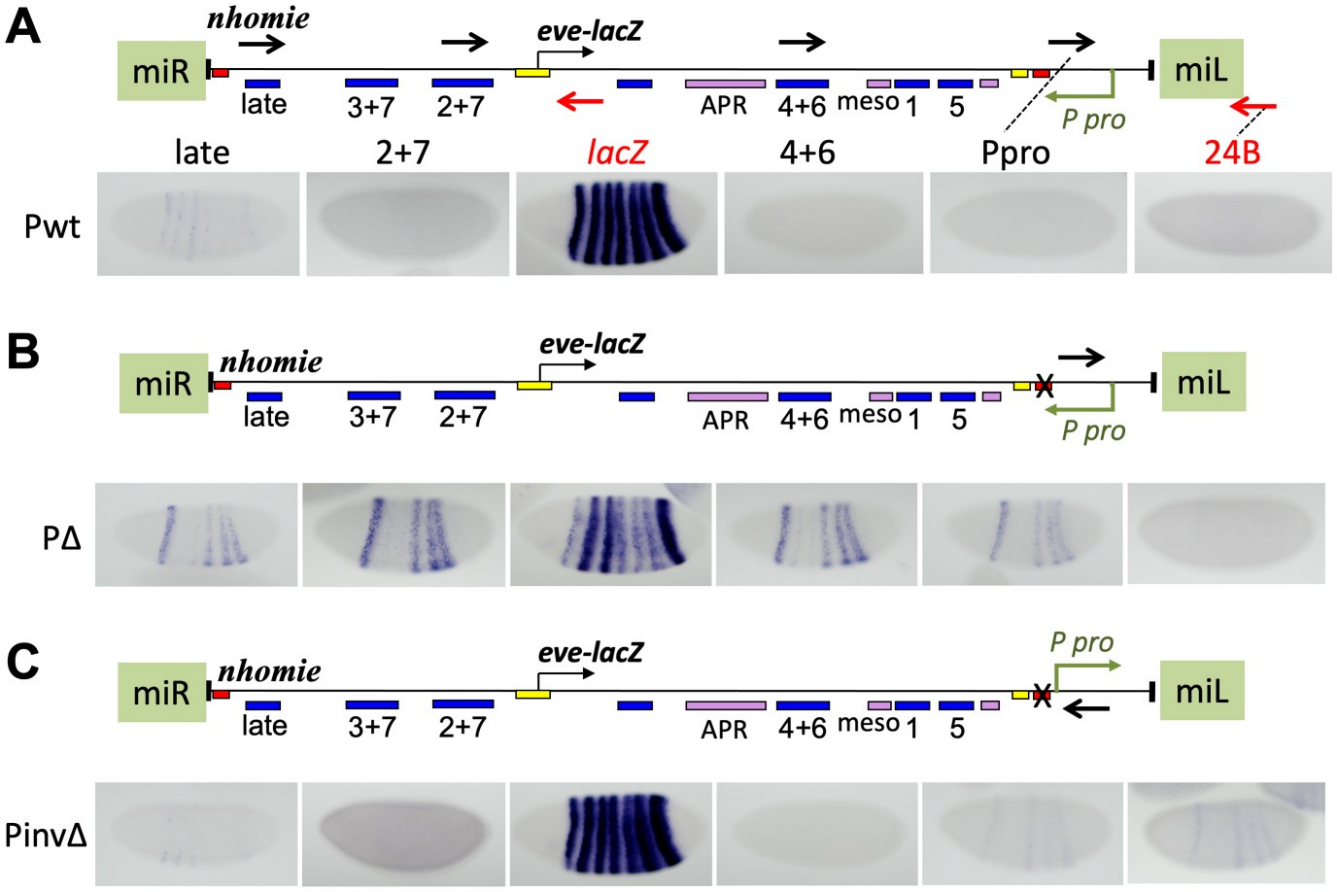
Inserting a flanking P-element promoter induces non-coding transcription, and if it causes read-through, repression of expression from the *eve* promoter. *eve-lacZ* and non-coding RNA expression in stage 5 embryos from *eve* pseudo-loci flanked by a 5’ P-element promoter (*P-pro*), inserted in the orientation diagrammed, at a MiMIC site (cytological location 24B1). The 3 loci (map above each one) differ only in the presence (**A**, Pwt) or absence (**B**, PΔ and **C**, PinvΔ) of *homie* (red box near *P-pro*), and in the orientation of *P-pro* (pointing to the left in A and B, and to the right in C). Positions and orientations of the RNA probes are shown as black and red arrows in the map in A (and in B and C for the probe closest to *P-pro*). Embryos were stained with the probes listed above in A, using either black lettering corresponding to black arrows in the map (detecting transcription from right to left), or red lettering corresponding to red arrows (detecting transcription from left to right).

The above experiments implicate the flanking P-element promoter in causing *eve-lacZ* repression when the intervening insulator is removed. What are the mechanisms behind this? It is possible that the read-through itself disrupts *eve* enhancer activity, as read-through from both P-element [45] and UAS-driven promoters has been seen to affect gene expression [44]. In the case of read-through coming from downstream of *eve-lacZ* (for example, in Fig 1B and 1D), continuous transcription would produce long anti-sense RNA, which might also cause RNAi-mediated degradation of *eve-lacZ* RNA. Another possibility is that promoter competition between the *eve* and P-element promoters reduces *eve-lacZ* expression. In order to test whether promoter competition is sufficient for repression, we introduced another set of transgenes into the MiMIC attP site (used in S4 Fig) that showed little effect from the surrounding chromatin environment. For the following experiments, the *TER94* portion of the pseudo-locus was removed to reduce the number of promoters involved. The construct without the *TER94* promoter expressed *eve-lacZ* similarly to the one with the *TER94* promoter, in both orientations of the transgene in the chromosome (S4B Fig compared to Fig 1B and 1C, "wt"). The P-element promoter was then added to the construct (Fig 4A, "P pro" on the map). In the “wild-type” construct, the P-element promoter transcribes toward the *eve* pseudo-locus, and *homie* is intact (Fig 4A, “Pwt”). In the second construct (Fig 4B, “PΔ”), *homie* was replaced by phage λ DNA. In the third construct, the P-element promoter was turned around relative to the “PΔ” construct, so that it transcribes away from the *eve* pseudo-locus, and *homie* is not present (Fig 4C, “PinvΔ”). In Pwt, there is no expression pattern detected in enhancer regions (Fig 4A, Pwt, probes 2+7, 4+6, Ppro, 24B, similar to that shown in Fig 3A), except for the same faint expression described above in the “late” region, which is independent of the P-promoter (Fig 4A, Pwt, “late” probe, similar to that seen in Fig 3A and S2 Fig; this expression, which is stronger in stripes 1, 2, 3, and 7 and weaker in stripes 4, 5, and 6, is more clearly seen in this insertion site than in the others). When *homie* is deleted (PΔ), stripes 1, 4, 5, and 6 were observed in the enhancer regions (Fig 4B, PΔ, probes “late”, 2+7, 4+6), as well as just downstream of the P-element promoter (“Ppro” probe). In PinvΔ, there is again expression in stripes 1, 4, 5, and 6 downstream of the P-promoter, which is now pointing away from the *eve* promoter (Fig 4C, PinvΔ, probes Ppro and 24B). As expected, no expression pattern was observed in the 2+7 or 4+6 region (Fig 4C, PinvΔ, probes 2+7, 4+6), while the late region showed the same P-promoter independent, faint expression in stripes 1, 2, 3, and 7. Probes recognizing the opposite strand did not show any expression pattern (S5 Fig, probes (late)R, (2+7)R, (4+6)R). Again, cDNA produced from specific primers showed that there are continuous transcripts from enhancer to enhancer (S3B Fig), consistent with the expression patterns being attributable to long non-coding RNAs initiated at the P-promoter. Quantification by RT-qPCR showed the expected trends, where transcript levels are elevated in PΔ lines relative to levels in lines that do not have the P-promoter pointing toward the *eve* promoter (S6 Fig, PΔ vs. wt, *yw*, and PinvΔ).

If repression of *eve-lacZ* is caused by transcriptional read-through, then *lacZ* RNA would be reduced in PΔ, but not in PinvΔ. If promoter competition is the cause, then *lacZ* RNA would be reduced in both PinvΔ and PΔ. In fact, *lacZ* RNA is reduced in PΔ, but not in PinvΔ. Therefore, *eve-lacZ* repression requires transcriptional read-through, while promoter competition alone is insufficient. This is the case even though when read-through is occurring, the enhancers driving the read-through expression are themselves subject to the read-through. We also cannot rule out a contribution to *eve-lacZ* repression from read-through of the *eve* promoter (in addition to the enhancers), which could disrupt either initiation itself, or E-P communication. It is also possible that promoter competition plays a role, even though it is not sufficient by itself to cause significant repression (see Discussion).

In the case where read-through transcription is coming from downstream of *eve-lacZ*, we cannot rule out a contribution to repression by an RNAi-based mechanism. However, in the N5 orientation shown in Fig 1C and 1E, the P-promoter is upstream of the *eve* promoter, so that it does not produce anti-sense RNA in the *eve-lacZ* coding region. Yet, we observed repression of *lacZ* in stripe 3 and in the 7 late stripes (Fig 1C and 1E). Although in this situation, the repression of *eve-lacZ* cannot be caused by RNAi, quantifying a reduction in *lacZ* expression by RT-qPCR is not straightforward, since the *lacZ* region is included in transcripts initiated by both the P-promoter and the *eve* promoter. That is, detected *lacZ* RNA (S7 Fig) is produced by both *eve-lacZ* expression and transcription from the P-element promoter that is repressing *eve-lacZ*. Nonetheless, RT-qPCR showed that *lacZ* RNA is reduced both in Δ*homie* compared to wild-type (S7A Fig, same lines used in Fig 1B), and in Δ*nhomie* compared to wild-type, (S7B Fig, same lines used in Fig 1C). Therefore, while a contribution from RNAi cannot be ruled out when the P-promoter is downstream of the *eve* locus, transcriptional interference caused by read-through from the flanking P-promoter does, indeed, occur in the absence of an intervening insulator.

### Do insulators block transcriptional read-through?

**Fig 5:**
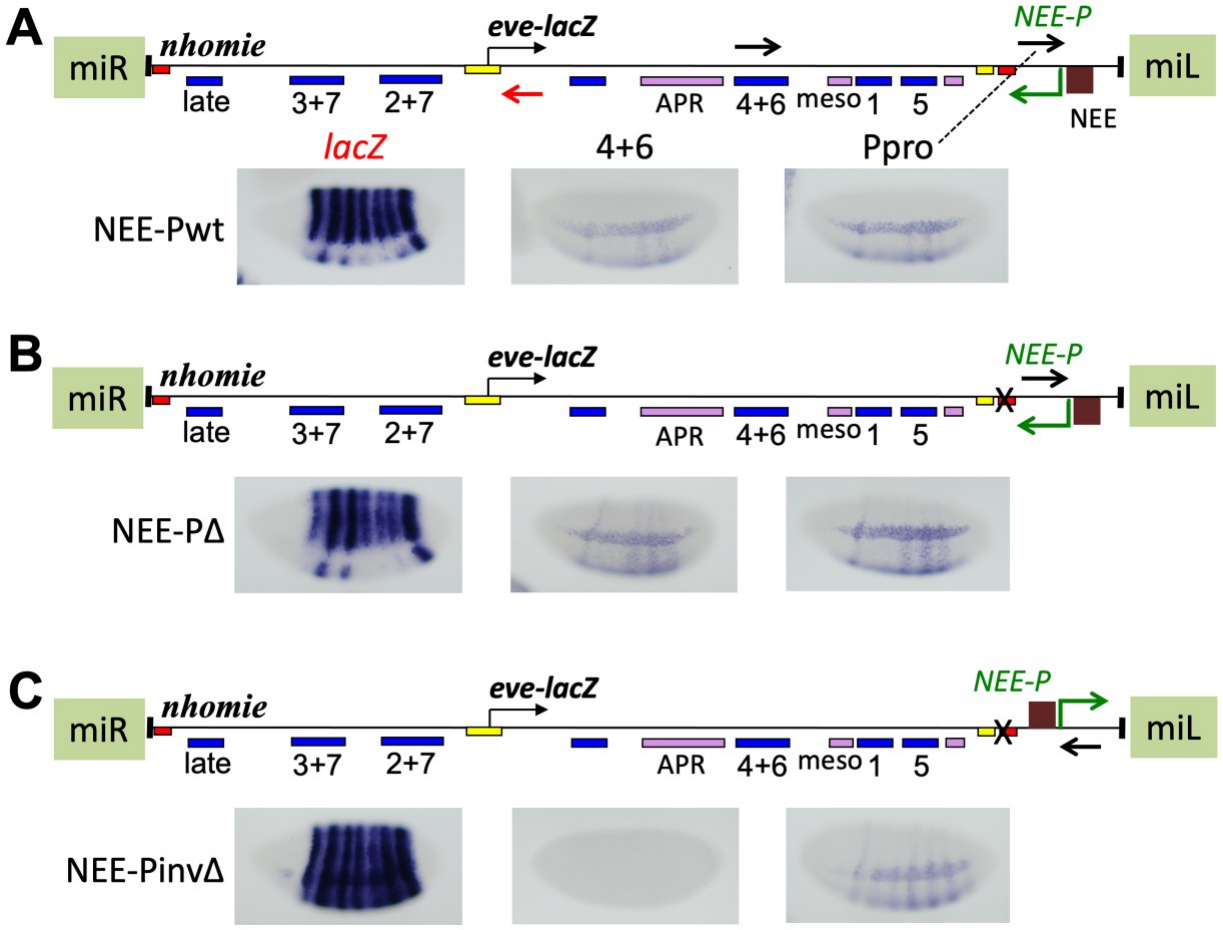
Driving a flanking P-element promoter with a heterologous enhancer induces non-coding transcription, and if it causes read-through, repression of the *eve* promoter. *eve-lacZ* and non-coding RNA expression in stage 5 embryos from *eve* pseudo-loci flanked by a 5’ P-element promoter and a neuroectodermal enhancer (*NEE*, brown square), inserted at the same MiMIC site as in Fig 4, and in the same orientation. The 3 loci (map above each one) differ only in the presence (**A**, NEE-Pwt) or absence (**B**, NEE-PΔ and **C**, NEE-PinvΔ) of *homie*, and in the orientation of the *NEE*, *P-pro* cassette (*NEE-P*, pointing to the left in A and B, and to the right in C). Positions and orientations of the RNA probes are shown as black and red arrows in the map in A (and in B and C for the probe closest to the P-promoter). Embryos were stained with the probes listed in A, using either black lettering corresponding to black arrows in the map (detecting transcription from right to left), or red lettering corresponding to the red arrow (detecting transcription from left to right).

An intervening insulator blocks communication between the *eve* enhancers and the P-element promoter. Can it block transcriptional read-through itself? To test this, a heterologous *rhomboid* enhancer, *NEE* [68], was added upstream of the P-element promoter (Fig 5), where it should activate the P-element promoter regardless of the presence or absence of *homie*. In the “NEE-Pwt” control, the P-element promoter is expressed in the *NEE* pattern of two ventrolateral “racing stripes” running anterior to posterior (Fig 5A, "NEE-Pwt", probe "Ppro"). In addition, early *eve* stripes 1, 4, 5, and 6 are seen between the two *NEE* stripes on the ventral side of the embryo (Fig 5A, “NEE-Pwt”, probe “Ppro”). However, the *eve* stripe pattern is not seen outside of this ventral region. This may be due to the action of NEE-bound activators in the ventral region that help to generate the racing stripes, acting in combination with activators bound to the *eve* enhancers that generate the *eve* stripes, to overcome enhancer blocking by *homie*. This same composite expression pattern was seen with a probe of the *eve* enhancer region (Fig 5A, probe “4+6”), suggesting that read-through from the P-element promoter is not significantly blocked by *homie*, regardless of which enhancers drive it. Wherever P-element transcripts were seen, *eve-lacZ* was repressed (Fig 5A, probe "*lacZ*"). When *homie* was removed, stripes in the ventral region became stronger, and the same stripes were also visible in the lateral and dorsal region, consistent with the removal of *homie*’s enhancer blocking activity (Fig 5B vs. Fig 5A). Again, *eve-lacZ* was repressed wherever these transcripts were present (Fig 5B). When the P-element promoter was turned around (with *homie* still not present), *NEE*-driven transcript expression was somewhat reduced, but the pattern of expression was the same as with the original orientation (Fig 5C, probe “Ppro”). However, consistent with our previous conclusion that repression is due primarily to read-through, and not to promoter competition, there was no detectable repression of *eve-lacZ*. In fact, *eve-lacZ* was also enhanced in the NEE pattern (Fig 5C, probe "*lacZ*"), showing that the “potentially competing” promoters are sharing NEE enhancer activity in the absence of *homie*. Interestingly, when there is read-through of the *eve* promoter, *lacZ* is not expressed in the *NEE* pattern, suggesting that read-through of the *eve* promoter region is sufficient for repression, since the *NEE* itself is not experiencing read-through (Fig 5B). In summary, driving read-through from a heterologous enhancer (the *rhomboid NEE*) causes disruption of *eve* expression, indicating that read-through is sufficient for repression. When *homie* is present, read-through transcription is driven primarily by the *NEE*, and *eve-lacZ* is repressed only where there is read-through. This shows that *homie* blocks only E-P interactions, not read-through transcription itself, at least of those transcripts initiated by the P-element promoter (which are highly processive, see below).

### Different promoters have distinct enhancer capturing capabilities, and different degrees of transcriptional processivity

**Fig 6:**
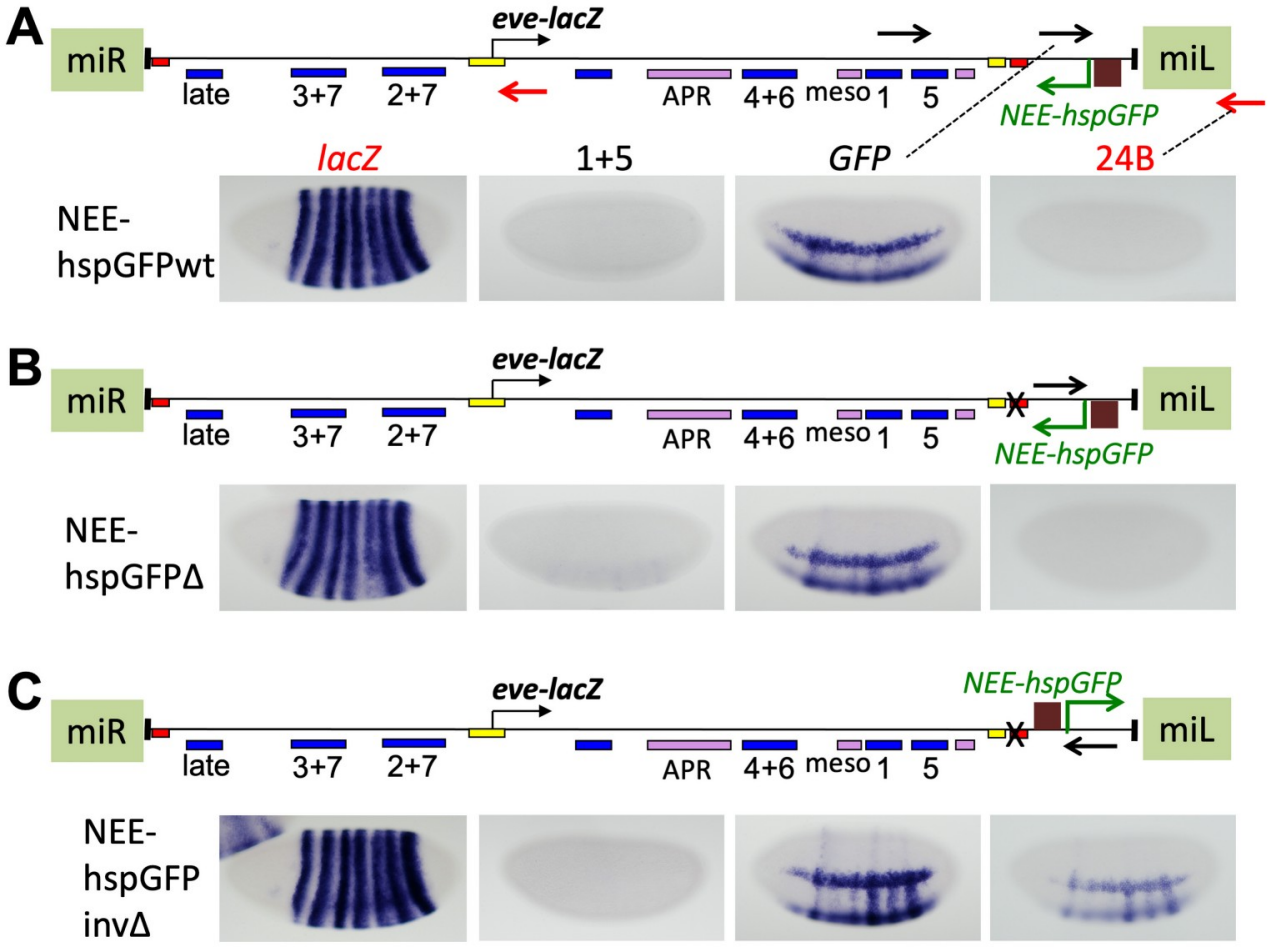
Inserting a flanking *hsp70* core promoter induces non-coding transcription that is not processive, and causes little repression of the *eve* promoter. *eve-lacZ* and flanking *hsp*-driven RNA expression in stage 5 embryos from *eve* pseudo-loci flanked by an *NEE*-*hsp70*-*GFP* cassette, inserted at the same MiMIC site as in Fig 4, and in the same orientation. The 3 loci (map above each one) differ only in the presence (**A**, NEE-hspGFPwt) or absence (**B**, NEE-hspGFPΔ and **C**, NEE-hspGFPinvΔ) of *homie*, and in the orientation of the *NEE*-*hspGFP* cassette (pointing to the left in A and B, and to the right in C). Positions and orientations of the RNA probes used are shown as black and red arrows in the map in A (and in B and C for the *GFP* probe). Embryos were stained with the probes listed in A, using either black lettering corresponding to black arrows in the map (detecting transcription from right to left), or red lettering corresponding to red arrows (detecting transcription from left to right).

To examine the effects of promoter specificity on the actions of flanking promoters in this system, we turned to the *hsp70* core promoter [66], which has been used frequently as a “general” regulated promoter in transgenic reporters [69]. To this end, we used it to drive a GFP reporter gene without a polyA signal, activated by the *NEE* (*NEE-hspGFP*). With *homie* in its normal place in the pseudo-locus, GFP transcripts are expressed in a similar pattern to the transcripts detected in the comparable construct with the P-element promoter, NEE-Pwt (Fig 6A, *GFP* probe; compare the pattern to Fig 5A, probe “Ppro”). When *homie* is removed, the *eve-*like stripes in the ventral region are a bit stronger, relative to the *NEE*-driven “racing stripes”, than with *homie* (Fig 6B, compare to Fig 6A, both with *GFP* probe), consistent with the loss of enhancer blocking activity. However, *NEE*-driven expression is almost undetectable in the *eve* regulatory region, with only very faint *eve* stripes seen on the ventral side (Fig 6B, probe 1+5). Reflecting this weak “read-through” expression pattern, *eve-lacZ* is repressed only in the ventral-most part of stripe 5 (Fig 6B, *lacZ* probe). When this *NEE-hspGFP* cassette is turned around to read away from *eve-lacZ*, *NEE*-driven expression of *GFP* transcripts, as well as strong ventral *eve* stripes with weak dorsal/lateral stripes, are seen (Fig 6C, probes *GFP* and 24B). Consistent with read-through being required for repression, *eve-lacZ* is expressed similarly to wild-type (Fig 6C, *lacZ* probe), except that some *NEE*-driven expression is also seen. It is likely that *homie* blocks this E-P communication in the “wt” construct (Fig 6A, *lacZ* probe). These data suggest that the *hsp70* core promoter may not respond as well to the *eve* enhancers as does the P-element promoter, at least at distances of several kb. They also suggest that the *hsp70* core promoter initiates transcription that has low processivity, relative to that initiated by the P-element promoter, when driven by the same enhancers.

**Fig 7:**
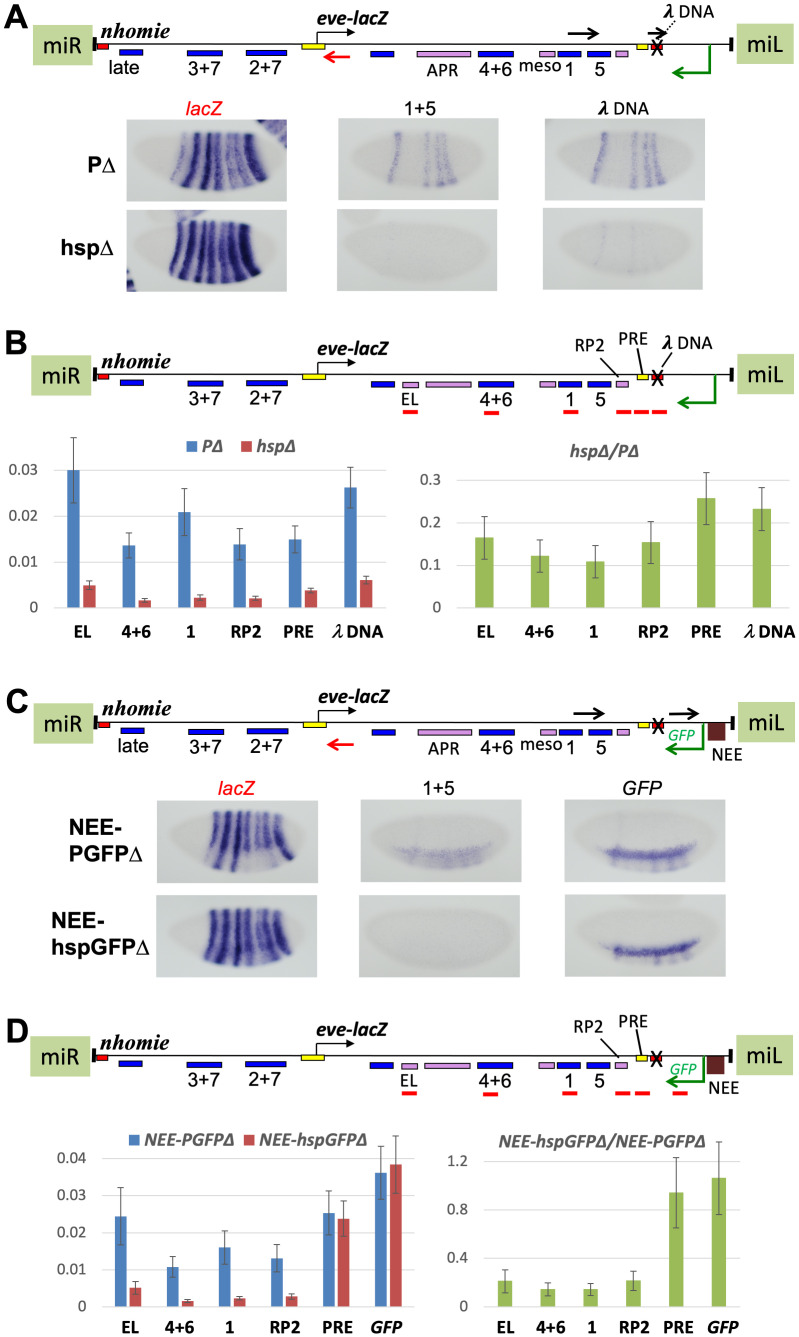
Processivity of read-through transcription from flanking promoters correlates with degree of repression of the *eve* promoter. Transgenes are at the same MiMIC site, and in the same orientation, as in Fig 4. All loci have *homie* replaced with phage λ DNA (refer to map above each one). **(A)** *eve-lacZ* and flanking promoter-driven RNA expression in stage 5 embryos. Positions and orientations of the RNA probes used are shown as black and red arrows in the map. Images are labeled (above) with black lettering corresponding to black arrows, or red lettering corresponding to red arrows. **Top row**: transgene with P-element promoter (PΔ, also used in Fig 4B). **Bottom row**: transgene with *hsp70* core promoter (hspΔ). Note that neither promoter has either the *NEE* or a *GFP* coding region. **(B**) Quantification of total RNA (normalized to *RP49* RNA) from the indicated enhancer regions (probed regions are shown as red bars in the map) in the PΔ and hspΔ lines. Averages with standard deviations of 4 biological samples each from hspΔ and PΔ are graphed on the left, and the ratios of average signals (with standard deviations) are graphed on the right. **(C**) Same as in A, except that the both promoters have the *NEE* and a *GFP* coding region (but no polyA signal). **(D**) Same as in B, except that the promoters are as described in C.

In order to eliminate the possibility that the *hsp70* promoter’s response to *eve* enhancers is weak due to competition with the *NEE*, we removed the *NEE*, as well as the GFP coding region. Thus, this construct is equivalent to PΔ, but with the *hsp70* core promoter replacing the P-element promoter (Fig 7A map). To compare activities of the two promoters directly, we used a probe to the *λ* sequence used to replace *homie*. The “*λ* DNA” probe showed expression driven by the P-promoter in *eve* stripes 1, 4, 5, and 6 (Fig 7A, PΔ). However, simultaneously stained embryos showed much fainter stripes driven by the *hsp70* core promoter (Fig 7A, hspΔ, probe "*λ* DNA"). Consistent with this difference in transcription levels, *eve-lacZ* expression was not noticeably repressed in hspΔ, while it was repressed in PΔ. RT-qPCR analysis showed that RNA expression at the enhancer regions from the *hsp70* promoter are 10–30% of those from the P-element promoter (Fig 7B, *hspΔ/P*Δ). These data suggest that the *hsp70* core promoter is not activated as well by the *eve* enhancers as is the P-promoter (however, see Discussion).

In order to test more directly whether transcription initiated at the P-promoter has higher processivity than that initiated at *hsp70* core, both NEE-PGFPΔ and NEE-hspGFPΔ (both of which have the *NEE* and the GFP coding sequence without a polyA site) were stained with probes located at different distances downstream. The promoter-proximal probe showed comparable levels of transcripts in both (Fig 7C, NEE-PGFPΔ and NEE-hspGFPΔ, probe "*GFP*"). In contrast, by the time transcription reached the stripes 1+5 enhancer region, expression was much lower in NEE-hspGFPΔ than in NEE-PGFPΔ (Fig 7C, probe "1+5"). RT-qPCR analysis showed a similarly high level of transcripts at promoter proximal *GFP* and PRE regions in NEE-hspGFPΔ and NEE-PGFPΔ (Fig 7D, *GFP* and PRE primer sets). However, consistent with the levels seen in Fig 7C, when the transcripts reach the RP2 neuronal enhancer and beyond, the transcription levels are reduced for both, but much more so for those initiated at the *hsp70* promoter (Fig 7D, RP2, 1, 4+6, and EL primer sets; note that primer efficiencies can vary, so that comparisons between primer sets are not as quantitatively valid as are the ratios with a given primer set, which are graphed on the right). These data indicate that the processivity of transcription initiated at the P-element promoter is considerably higher than that initiated at the *hsp70* core promoter, when driven by the same enhancers.

### An intervening poly-adenylation signal only partially blocks transcriptional read-through

**Fig 8:**
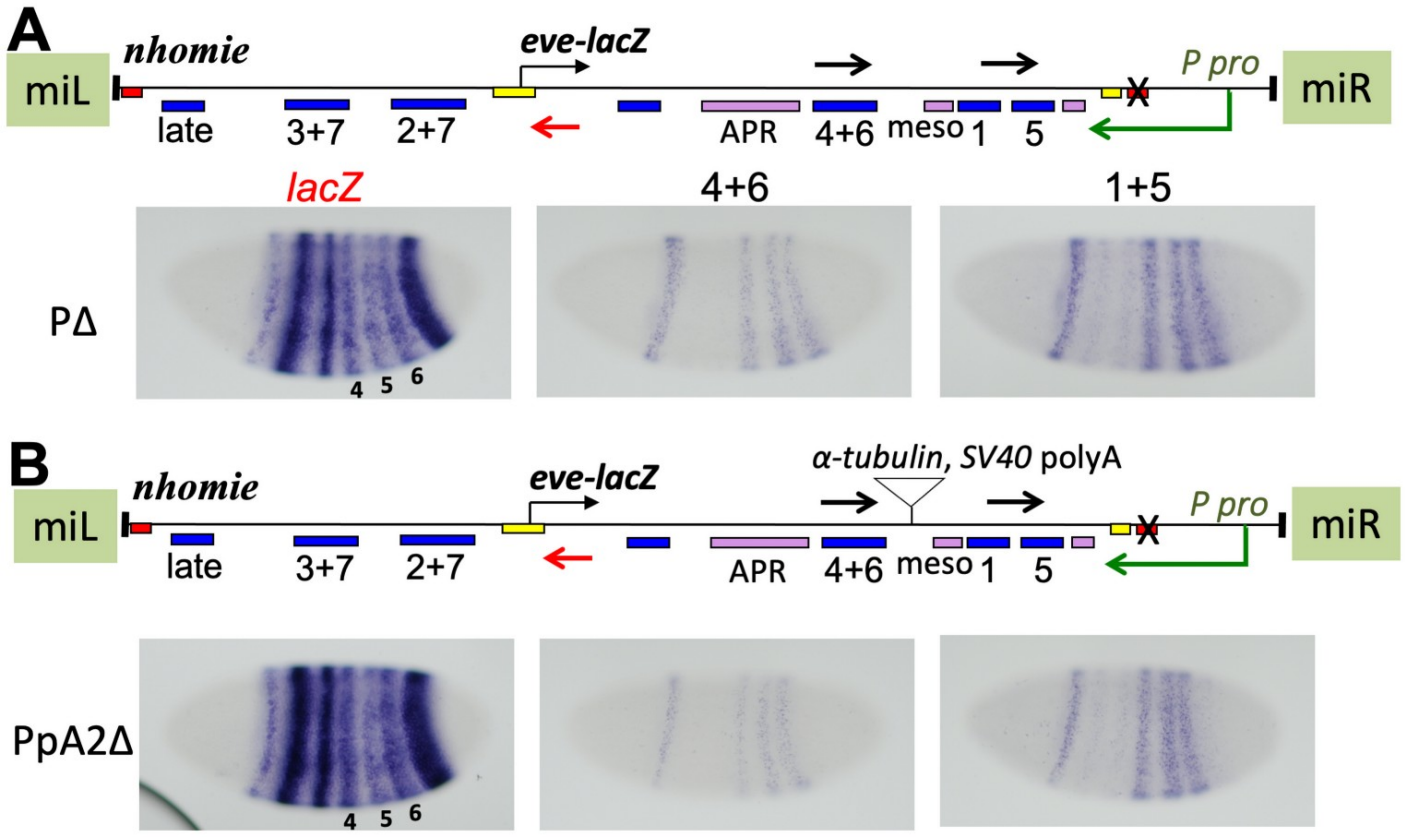
Insertion of polyA-addition signals weakly attenuates read-through transcription from a P-element promoter and correspondingly reduces repression of the *eve* promoter. *eve-lacZ* and flanking P-element promoter-driven RNA expression in stage 5 embryos from *eve* pseudo-loci, inserted at the same MiMIC site as in Fig 4, but in the opposite orientation. All loci have *homie* replaced with phage λ DNA (refer to maps). Positions and orientations of the RNA probes used are shown as black and red arrows in the maps. Images are labeled with black lettering corresponding to black arrows, or red lettering corresponding to the red arrow. The loci in **A** (PΔ, also used in Fig 4B) and **B (**PpA2Δ), differ only in the insertion of a tandem array of polyA-addition signals, one from *α-tubulin* and two from *SV40*, at the position shown in the map in B.

In order to test whether the transcriptional read-through effect is due more to read-through of the *eve* promoter or the enhancers (and/or disrupting E-P interactions), the commonly used *α-tubulin* polyA signal [64] was inserted between the 4+6 and mesodermal enhancers in the PΔ construct (which carries the P-element promoter without *homie*, used in Figs 4B and 7A). If read-through is inactivating only the *eve* promoter, stopping transcription before it reaches the promoter would rescue all *eve-lacZ* expression. If read-through is inactivating only enhancers, stripes 1 and 5 would still be repressed. Unexpectedly, the transcription pattern beyond the *α-tubulin* polyA signal did not diminish much at the 4+6 enhancer region, indicating that the polyA signal did not effectively stop read-through (S8A and S8B Fig, probe 4+6). However, the introduction of the polyA signal did cause a slight increase in *eve-lacZ* expression, most noticeably in stripes 4 and 6 relative to 5 (compare intensity of stripes 4 and 6 to stripe 5 with *lacZ* probe in S8A Fig vs. S8B Fig), suggesting that the polyA signal reduces read-through from the P-element promoter a bit, which results in partial rescue of stripes driven by enhancers downstream of the polyA site. This, in turn, suggests that read-through of enhancers reduces expression. In order to try to get a clearer result, we introduced a pair of tandem polyA signals from *SV40* [65], in addition to the *α-tubulin* polyA signal, in the PΔ construct (Fig 8A and 8B). Again, we did not observe a clear decrease in expression beyond the polyA signals (Fig 8A and 8B; probe 4+6 vs. 1+5). And again, *lacZ* expression was only weakly rescued in stripes 4 and 6 relative to stripe 5 (Fig 8, probe *lacZ*, compare intensity of stripes 4 and 6 to stripe 5 in A vs. B). There may also be an increase in expression of the other stripes relative to that of stripe 5, but the effect remains subtle. Quantitation of transcript levels by RT-qPCR showed that introduction of the polyA signals caused lower levels from the P-promoter overall (S9A Fig). The ratio of transcript levels in PpA2Δ to those in PΔ was 82% and 71% at the λ DNA and the stripe 1 enhancer, respectively (both upstream of the polyA signals). Downstream of the polyA signals, this ratio was decreased to 58% and 56% at the stripe 4+6 and 3+7 enhancers, respectively, suggesting again that the polyA signals may be causing termination of a small fraction of P-promoter initiated transcripts. Finally, we tested the NEE-PGFPΔ construct with and without the same polyA signals to see if heterologous enhancer-driven transcription from the P-element promoter is affected in the same way, with similar results (S8C and S8D Fig). As before, transcript levels appeared to be slightly reduced at the 4+6 enhancer relative to the 1+5 enhancer region, and *lacZ* expression in stripes 4 and 6 was rescued just noticeably relative to expression in stripe 5, again suggesting that enhancer read-through may have a repressive effect on expression.

## Discussion

### A myriad of insulator functions in gene regulation

It is widely appreciated that removing insulators can cause the influences of both enhancers and silencers to spread inappropriately along the chromosome, changing gene expression in both flanking regions [1]. What have not been clearly described are the important secondary effects that changes in transcription can produce, one of the most direct of which is disruption of enhancer and promoter function by transcriptional read-through coming from outside the TAD. Here, we describe such an effect at the *eve* pseudo-locus, which we have analyzed in detail to show that promoter competition is not sufficient for the disruption of *eve-lacZ* expression that ensues when an insulator is removed. There are two main lines of evidence for this. First, turning the flanking promoter around causes loss of most or all of the repression. Second, read-through driven by an added enhancer is sufficient to disrupt expression driven by the other enhancers.

We further show that insulators do not block read-through *per se*, consistent with data previously reported [44]. Instead, insulators prevent enhancers within one locus from activating a flanking promoter, which, without the insulator, would disrupt enhancer action on the appropriate promoter. Such indirect effects may be common when insulator activity is compromised, as we have also found that the P-element promoter produces transcripts that read through not only an insulator, but also polyA addition signals from both *α-tubulin* and *SV40*, suggesting that significant read-through may be a common phenomenon, even though the relevant polyA site(s) is intact.

A recent study showed that TADs in Drosophila usually correspond to a single locus [40]. This suggests that enhancer-promoter interactions may be a major driving force underlying TAD-level architecture. We showed that removing either *homie* or *nhomie* allows a promoter outside the locus to interact with *eve* enhancers, and by reading toward the *eve* locus, capture their activity and repress the *eve* promoter. Similarly, a recent study showed that removing the Fub insulator in the BX-C caused collapse of the corresponding TAD boundary [31]. Our data suggest that different genes will react differently to inactivation of insulators, since each gene is in a different chromatin context. Thus, understanding the effects of removing insulators on individual genes may be as important as identifying the general effects of insulators.

### Transcriptional read-though in gene regulation

In this study, we showed that transcriptional read-through effectively represses *eve-lacZ* expression, and that *homie* does not block read-through. Is this an unusual case caused by a P-element promoter? Studies of the BX-C showed that long non-coding RNAs (ncRNAs) can read through insulators and affect gene regulation [47]. For example, the *iab-8* ncRNA extends for 92 kb in the posterior segments of the embryo where *abd-A* is expressed at a relatively low level, and is required for repression of *abd-A* in the central nervous system. Two repression mechanisms were suggested, a *trans* effect due to a microRNA produced from the *iab-8* ncRNA, and a second effect acting in *cis*. The *cis* effect is most likely transcriptional interference caused by transcription through *abd-A* [47]. The *iab-8* ncRNA extends through several *abd-A* regulatory modules containing early- and late-acting enhancers that are separated by insulators [70]. Consistent with our study, these insulators do not stop transcriptional read-through. However, the enhancers there also do not activate the *iab-8* ncRNA promoter, because the intervening insulators do block enhancer-promoter interactions. Taken together, these studies suggest that gene repression by transcriptional read-through is a likely outcome when insulator function is disrupted.

The *eve* enhancers can activate a flanking P-element promoter in the absence of an intervening insulator. In particular, when *homie* was present, a flanking P-element promoter was not detectably activated by the *eve* enhancers. On the other hand, we did observe some weak stripe expression when *nhomie* was the intervening insulator. This expression was, as expected, much stronger when *nhomie* was removed (Fig 3B, "wt" and "Δ*nhomie*"). These data show that *nhomie*’s enhancer blocking activity is somewhat weaker than that of *homie*. More generally, our results suggest that while blocking of *cis* effects along chromosomes by insulators may not be absolute, inappropriate interactions become weak enough when they are present so that genes can be considered separate functional genetic units, in most cases.

The transcripts we detected at various enhancers throughout the locus are continuous from the flanking P-element promoter, and are not short non-coding RNAs (e.g., eRNAs). One line of evidence for this is that the RNAs are unidirectional, since they were not detected by *in situ* hybridization using probes against the opposite strand (Fig 3A, S2 and S5 Figs). In addition, RT-qPCR analysis of cDNA produced from specific primers showed that the RNAs are continuous from one enhancer to the next (S3 Fig). This confirms that it is transcription initiated from the flanking promoter in response to *eve* enhancers that is responsible for disrupting *eve* promoter activity when an intervening insulator is removed.

What are the mechanisms of transcriptional interference by read-through? One possibility is inactivation of enhancers, since the transcriptional machinery must at least transiently displace DNA binding proteins as it passes. How strongly such displacement affects promoter activity is expected to depend on the dynamics of the E-P interactions in any given case. We attempted to see if this is the case for *eve* enhancers by adding polyA signals to terminate read-through transcription between enhancers. We were surprised to find that the read-through transcription was not effectively stopped even by a tandem array of 3 polyA-addition signals, one from *α-tubulin* and 2 from *SV40*. We did see evidence of weak attenuation, accompanied by correspondingly weak rescue of a downstream enhancer’s activity on the *eve* promoter (Fig 8 and S8 Fig, compare stripes 4+6 to stripe 5 within individual embryos). Because the effect is small, we can only suggest that read-through of enhancers may be sufficient for transcriptional interference. It is possible that most of the effect we see here is due to read-through of the *eve* promoter (see below). Consistent with the P-element promoter initiating highly processive transcription, wild-type P-elements have two polyA signals. The first does not stop transcription completely, resulting in two sizes of transcript [71,72].

In light of the fact that many genes have enhancers in their introns, it may seem implausible that enhancers would be inactivated by read-through. However, such an effect would only be expected to reduce the rate of transcription, since a reduced rate of transcription would result in a reduced amount of repression, and eventually a steady-state balance would be reached. Since most genes are not expressed at very high levels, such an effect is entirely plausible.

Read-through driven by a heterologous enhancer inserted upstream of the P-promoter (Fig 5, *NEE* enhancer), so that the enhancer itself is not being read through, seems sufficient to repress *eve* promoter activity. Although we cannot completely rule out that the repression we observe is due to enhancer read-through, it is more likely the result of disruption of both enhancers and the *eve-lacZ* promoter. Thus, our data are consistent with the idea that enhancer-promoter interactions are disrupted by read-through of either interacting element.

In the case where the flanking promoter that is responsible for read-through (and for the resulting transcriptional interference) is downstream of the *eve* locus, and reads through the coding region as well as the *eve* promoter, another possible mechanism for the observed repression is RNAi (Figs 3A and 4B). Although we cannot rule this out as a contributing mechanism when the read-though is coming from downstream of *eve-lacZ* (e.g., Fig 1B and 1D), when read-through is coming from upstream, no anti-sense RNA is produced. In such cases (e.g., Fig 1C and 1E), we observed significant repression of *lacZ* in the pattern of the non-coding RNA produced from the flanking promoter, just as we did when the flanking promoter was downstream (S7 Fig). This is the case even though the RNA transcribed from the *lacZ* coding region is the sum of transcripts initiating at both the *eve* promoter and the flanking promoter that is responsible for the repression. This suggests that the transcriptional interference observed in this study is quite “efficient”, producing a reduction in *eve* promoter-initiated transcripts that is greater than the number of read-through transcripts causing the repression. Thus, our data show that read-through can cause repression by transcriptional interference without the involvement of anti-sense RNA.

It was reported that a P-element inserted into the BX-C caused segment A1 to A2 transformation, and this effect was ascribed to a P-promoter that produced a long ncRNA [45]. Thus, a P-element promoter in other contexts can generate read-through that disrupts gene expression. Such effects are not limited to P-element promoters. For example, it was shown that ncRNAs initiated in the *bxd* region of the BX-C repress *Ubx* expression in *cis* by transcriptional interference [73]. More complex effects of read through are also known to occur. For example, one of the promoters contained within the *scs* insulator generates read-through transcription that can inactivate a PRE [46]. These results, along with our observation that removal of insulator function allows flanking promoters to capture enhancers that they would not otherwise have access to, suggest that preventing read-through caused by inappropriate enhancer capture may be a common function of many insulators throughout the genome.

There are 3 ncRNAs in the *eve* locus, according to Flybase [74]. One is initiated just 5’ of *TER94* (lncRNA:CR45324); however, this initiation site is not present in any transgenes used in Fig 4 or later. Another (asRNA:CR43948) begins just 5’ of the *eve* coding region. This initiation site is not present in any of our constructs, since we replaced the *eve* coding region with that of *lacZ*. Furthermore, according to Flybase, neither of these RNAs is expressed at any of the stages used in this study. The initiation site of the 3^rd^ ncRNA (lnc-RNA:CR46455) is present in our transgenes. It is near the 5’ end of the *eve* locus, within the late 7-stripe element, and is transcribed toward the *eve* coding region. This transcript should be recognized by our “(late)R” probe, but we did not detect any pattern with this probe (Fig 3A, Δ*homie*). RT-qPCR showed very low expression in this region in our control *yw* fly strain (Fig 2A and S6 Fig). Therefore, it is extremely unlikely that this ncRNA is involved in any of our results. Interestingly, *in situ* hybridization with “late” probe detected faint 1, 2, 3, 7 stripe expression in our transgenic lines (Fig 3A, *wt*; Fig 4, Pwt and PinvΔ; S2 Fig, Δ*nhomie*), but not in embryos without a transgene (S2 Fig, *yw*). The “late” probe detects transcripts coming from downstream of the “late” enhancer. However, the pattern is not the one expected for transcripts coming from the downstream P-element promoter in Figs 3A and 4 (which would be in stripes 1, 4, 5, 6, driven by enhancers in the 3’ region). Instead, this weak expression is stronger in stripes 1, 2, 3, and 7 (and so is likely driven by enhancers 5’ of the *eve* start site). Therefore, it is likely that there is another ncRNA start site downstream of the “late” stripe enhancer, but 5’ of the *eve* transcription initiation site. Nonetheless, this weak expression does not correlate with any of the results in this study, and so we conclude that it is unlikely to be consequential for either *eve* or *eve-lacZ* expression.

### Promoter specificity and processivity

When a core *hsp70* promoter is used in place of the P-element promoter, read-through transcripts are weakened, as is repression. This suggests that the P-element promoter is relatively efficient at responding to *eve* enhancers (Fig 7A). In an earlier study, we reported that the *eve* upstream promoter region can facilitate *homie-homie* long-range interaction activity. Replacing the *eve* promoter with the *hsp70* core promoter reduced this activity [7]. This is consistent with the idea that the efficiency (or, more specifically, the stability) of enhancer-promoter interactions can affect the stability of long-range insulator interactions [75]. This illustrates the dynamic interplay between chromosome architecture and gene regulation, and the complex mechanisms by which insulators affect gene expression.

Importantly, little reduction in intensity is seen between transcripts detected at different distances downstream of the P-element promoter, suggesting that read-through is processive (Figs 3A and 4). In contrast, when the *hsp70* promoter is used, while there are easily detectable transcripts near the start site, they are almost undetectable a few kb downstream (Fig 6). This is true when expression is driven by either the *eve* enhancers or the *NEE* (Fig 7). For *NEE*-driven expression (Fig 7D), analysis by RT-qPCR showed a level of expression from the *hsp70* promoter comparable to that from the P-promoter at 0.7 and 1.7 kb from the start site (at the positions of the *GFP* and PRE primer sets). However, the level of transcripts from the *hsp70* promoter dropped relative to those from the P-promoter between the position of the 3’ PRE and the RP2 neuronal enhancer (2.3 kb from the start site). We consider two possibilities for this apparent termination of transcripts from the *hsp70* promoter. First, the *eve* locus is known to be a Polycomb domain, which is characterized by the histone methylation mark H3K27me3 [76,77]. This repressive chromatin environment might affect processivity of transcription, causing the less processive transcription from *hsp* to terminate more frequently than that from the P-promoter. A second possibility is that transcription from *hsp* is responding to a termination signal where ncRNA CR45324 ends, which is between the RP2 enhancer and the 3’ PRE. (This ncRNA is initiated within the 1^st^ exon of *TER94*.) In summary, these data suggest that the P-promoter drives more processive transcription in combination with a variety of enhancers than does the *hsp70* core promoter. This processivity allows RNAPII to read through *eve* enhancers located many kb away, when there is an intervening insulator, and even through and far beyond multiple polyA signals. It will be interesting to investigate how different promoters, activated by the same enhancers, cause differences in RNAPII processivity.

As discussed above, when polyA-addition signals were introduced downstream of the flanking P-promoter, RNAPII did not efficiently terminate transcription. Two models for how transcription termination occurs have been proposed [78]. A recent study in *C*. *elegans* showed that the promoter can influence which mode is used [79]. Further study will be required to determine whether the two promoters we have compared here use either of these modes of termination, as well as to understand how some promoters generate transcription that fails to terminate downstream of polyA signals.

### Promoter competition vs. transcriptional interference by read-through

In most cases we examined, there is a close correlation between the amount of transcription driven by the competing P-element promoter and the degree of repression of *eve-lacZ*. This is consistent with either promoter competition or transcriptional interference as a mechanism of repression. However, when the P-promoter is turned around so that read-through is eliminated, repression is strongly reduced, showing that read-through is necessary for the repression (Fig 4). Furthermore, read-through driven by a heterologous enhancer is sufficient for repression, implicating transcriptional interference caused by read-through as the primary mechanism of repression (Fig 5). Thus, our data are consistent with there being little promoter competition at work, which in turn suggests that enhancer-promoter interactions are highly dynamic while not approaching saturation. This scenario allows both promoters to be activated in an *eve* stripe pattern without significant competition.

There is an important caveat to this view, however. When the P-element promoter is turned around, its level of transcription appears to be reduced. If this is indeed the case, it suggests another interesting possibility, one that involves significant promoter competition. If we suppose that *eve* E-P interactions are more stable than those that occur between *eve* enhancers and the heterologous P-promoter, we might expect that *eve* enhancers would preferentially interact with the *eve* promoter when a competing P-promoter is brought into the same TAD (either by insertion of the P-promoter or by removing an intervening insulator). However, if transcription from the P-promoter reads toward the *eve* locus, and if that transcription interrupts stable *eve* E-P interactions, freeing the enhancers to interact with the P-promoter, it could facilitate enhancer capture by the P-promoter, allowing it to better compete for the enhancers. This in turn would boost P-promoter activity at the expense of *eve* promoter activity, repressing *eve*. This mechanism might also explain why the *hsp70* core promoter is not activated as well by *eve* enhancers as is the P-promoter, since transcription from it can’t effectively release the *eve* E-P contacts, due to its low processivity. Additional work will be required to understand the dynamics of these interactions, and to tease out how much of the repression that we see is due to transcriptional interference caused by read-through of enhancers, how much is due to transcriptional interference caused by read-through of the *eve* promoter, and whether promoter competition plays a significant role. These repression mechanisms are not mutually exclusive, and all can be a consequence of insulator removal.

## Methods

### Plasmids and transgene production

The original *eve* pseudo-locus constructs have been described previously [12]. A modified version which includes the complete *nhomie* insulator [19] was used in this study. The DNA fragment from –6.5 kb (instead of the –6.4 kb end point used in the original construct) to +166 bp relative to the *eve* TSS was fused to the *lacZ* coding region. The 3’ end of the *lacZ* coding region was fused to DNA from +1.3 to +11.4 kb, which includes the *eve* polyA signal, and extends into the 3^rd^ exon of *TER94*. The EGFP coding region, followed by the polyA signal of *α*–*tubulin*, was added at the 3’ end. The entire construct was placed between two oppositely oriented attB sequences [53]. Replacement of *homie* (from +9.3 to +9.8 kb) in these constructs by λ DNA or by heterologous insulators was described previously [12]. The original 5’ end point of the pseudo-locus at –6.4 kb was used as our *nhomie* deletion (described herein as Δ*nhomie*). The heterologous insulator assays of Fig 1F (including wt and Δ*homie* in that figure) were done in this Δ*nhomie* context. Exact end points are shown in S10 Fig. These transgenic vectors were inserted using φC31 RMCE [53] as described previously, into chromosomal *attP* sites that had previously been introduced into the Drosophila genome by P-element transgenesis [12]. The direction of each insertion was determined by PCR. Orientations of inserts are listed in the text as either the "H5 orientation", in which the 3’ (“*homie*”) end of the locus is close to the 5’ P-element end, or the "N5 orientation", in which the 5’ (“*nhomie*”) end of the locus is close to the 5’ P-element end. The *attP* sites used are those at cytological locations 95E5, for Figs 1B, 1C, 1F, 2, 3A, 3B and S3A Fig, and 23C4 for Fig 1D and 1E and S1 and S2 Figs.

To test the effects of transcriptional read-through, the following new transgenic vectors were made. Starting with the modified pseudo-locus described above (which includes *nhomie*), *TER94*-EGFP was removed, and the 3’ end was shortened to +9.8 kb, which is the 3’ end of *homie*. The *homie* sequence (from +9.3 to +9.8 kb) was then replaced by λ DNA. Modified forms of these with either “wt” or “Δ” in the name were used in the figures after Fig 4 and S5 Fig, except S7 Fig. Either the P-element promoter alone (*P*) [60–63], the P-element promoter with the *rhomboid* neuroectodermal enhancer (*NEE-P*) [68], or the *heat shock 70* core promoter [66] driving EGFP without a polyA signal (*NEE-hspGFP*) was then inserted at the 3’ end of each of these, in either orientation, as diagrammed in Figs 4–8. The following additional modifications were also made: the P-element promoter with the GFP coding region (without a polyA signal) driven by the *NEE* was inserted into the construct without *homie* to give NEE-PGFPΔ (used in Fig 7C and S8C Fig); an *α-tubulin* polyA signal [64] was inserted at +6621 nt in PΔ, between the stripes 4+6 and mesodermal enhancers, to yield PpAΔ (used in S8B Fig); an *α-tubulin* polyA signal [64] plus two *SV40* polyA [65] signals were inserted at +6621 nt in both PΔ and NEE-PGFPΔ to yield PpA2Δ (used in Fig 8) and NEE-PGFP-pA2Δ (used in S8D Fig), respectively. Each of these vectors was inserted into the MiMIC target site Mi{MIC}Drgx [MI04684] (cytological location 24B1) [67,74]. Orientations of the inserts are as noted in the figure legends. Relevant sequences are given in S10 Fig.

### RT-PCR

Total RNA was purified from 2–4 h embryos (or from 2.5–4 h embryos for S7B Fig), using an RNA purification kit (Roche Applied Science). cDNA was synthesized using the Transcriptor first strand cDNA synthesis kit with random primer, following the manufacturer’s protocol (Roche Applied Science) for Figs 2 and 7 and S1, S6, S7A and S9 Figs. For S3 Fig, specific primers for “late” or “EL” along with “RP49”-specific primer were used to make cDNA. Each biological sample was analyzed in triplicate by real-time PCR (Life Technologies, StepOnePlus) using SYBR Green Master Mix with ROX dye (Roche Applied Science). PCR data were analyzed with StepOne software, using the standard curve method. *RP49* was used as a reference gene, while *eve* was also used as a reference gene for *lacZ* expression in S7B Fig. Each experimental value was divided by the *RP49* value (and *eve* in S7B Fig) from the same sample. Primers used for RT-qPCR are given in S10 Fig.

### *In situ* hybridization

Embryos were collected and subjected to *in situ* hybridization as described previously [12]. Enhancer regions were cloned in vector pSP72 (Promega), then digested with appropriate enzymes to make labeled run-off transcripts using T7 or SP6 RNA transcriptase, with the DIG RNA labeling kit (Roche Applied Science). The two probes produced by T7 or SP6 recognize the same enhancer region, but different strands. The genomic regions used for probes are listed in S10 Fig. In order to reduce staining variability when lines were to be compared, *in situ* hybridization was performed in parallel at the same time. Representative embryos from the stained populations are shown.

## Supporting information

S1 Fig Non-coding RNA expression is increased by *eve* insulator removal. **(A, B)** transgenes in the H5 orientation (see Fig 1A) at site 23C4. **(C, D)** transgenes in the N5 orientation (see Fig 1A) at 23C4. **(A, C)** RT-qPCR quantification of total RNA (normalized to *RP49* RNA) from the indicated enhancer regions (probe locations shown as red bars below the map), in the *wt* and Δ*homie* transgenic lines (used in Fig 1D) in A, or in *wt* and Δ*nhomie* (used in Fig 1E) in C. Averages with standard deviations of 3 biological samples each are graphed. **(B, D)** The ratios of average signals (with standard deviations) from *wt* and Δ*homie* in B, and *wt* and Δ*nhomie* in D are graphed. Note the general trend toward a decrease in average signal moving away from the location of the 5’ P-element end (the right side in B and the left side in D).
(DOCX)

S2 Fig Patterns of non-coding RNA expression caused by *nhomie* removal. **Map**: Transgene construct, showing location of the 5’ P-element end, where read-through transcripts are initiated. **Images**: RNA *in situ* hybridization to embryos at stage 5 carrying Δ*nhomie* at 23C4 (used in Fig 1E). Probes are shown as either red arrows recognizing transcripts transcribed from left to right in the map, or black arrows recognizing transcripts transcribed from right to left. Labels use either black lettering corresponding to black arrows, or red lettering with R corresponding to red arrows.
(DOCX)

S3 Fig Non-coding transcripts are continuous across enhancers. cDNA primed in either the "late" or “EL” (green block arrows) enhancer regions were analyzed by qPCR using primers recognizing other enhancer regions (red bars below the map) (normalized to *RP49*, analyzed in parallel for each sample, using *RP49*-specific primers). **(A)** cDNA from *wt* and Δ*homie* transgenes at 74A2 (used in Fig 1B). **Left graph**: cDNA from "late" primer analyzed with “3+7” primer set. **Right graph**: cDNA from "EL" primer analyzed with “4+6”, “1”, and “wD5” primer sets. The "wD5" primer set was used to recognize cDNA representing the region upstream of the P-element promoter (a negative control). **(B)** Same as A, except that cDNA is from Pwt and PΔ transgenes (used in Fig 4A and 4B) at 24B1, and the “24B” primer set was used to recognize cDNA representing the region upstream of the P-element promoter (negative control).
(DOCX)

S4 Fig Insulator removal does not cause significant changes in expression in the absence of a flanking P-element promoter. **(A) Top**: Map of the *eve* pseudo-locus. miR and miL are Minos inverted repeats. **Bottom**: Expression of *eve-lacZ* from the pseudo-locus inserted into a MiMIC site at cytological location 24B1 detected by *in situ* hybridization. Both orientations of insertion are shown (left and right panels) at embryonic stages 5, 7, 11, 13, (as indicated), with either *nhomie* alone (Δ*nhomie*), or both *nhomie* and *homie* (Δ*nhomie*, Δ*homie*), deleted. **(B) Top**: Map of the *eve* pseudo-locus modified by removal of *TER94-GFP*. **Bottom**: Same as in A, using a line carrying this modified pseudo-locus.
(DOCX)

S5 Fig There is no apparent expression from upstream in the modified *eve* pseudo-locus at the 24B1 MiMIC site. Same as Fig 4, except probes recognizing the opposite strand are used. Positions of probes are shown as red arrows, and images are labeled with red lettering with R.
(DOCX)

S6 Fig Modified *eve* pseudo-loci show read-through transcription when a P-element promoter is added to the original constructs at the 24B1 MiMIC site. **Map**: The wt transgene with locations of PCR products used for transcript detection shown as red bars under the map. **(A)** Loci used in Fig 4A (wt), Fig 4B (*P*Δ), Fig 4C (*Pinv*Δ), and *yw* (without transgene; signals come only from endogenous *eve*) were subjected to RT-qPCR quantification of total RNA (normalized to *RP49* RNA) from the indicated enhancer regions. Averages (with standard deviations) of 4 biological samples each are graphed. Note that transcript levels are strongly increased throughout the locus in *P*Δ, but not in wt or *Pinv*Δ. **(B)** The ratios of average signals (with standard deviations) from *P*Δ and wt are graphed. Note the general trend toward a decrease in the relative *P*Δ signal moving away from the location of the P-element promoter. **(C)** Similar to A, except that the set of wt, *P*Δ, and *Pinv*Δ transgenes are inserted in the opposite orientation at the same MiMIC site, and averages (with standard deviations) of 5 biological samples each are graphed. **(D)** The ratios of average signals (with standard deviations) from *P*Δ and wt in C are graphed.
(DOCX)

S7 Fig Quantification of expression downstream of the *eve* promoter shows a significant reduction caused by removal of *nhomie*, even though this expression is a combination of that driven by the *eve* promoter and the upstream P-element promoter. RT-qPCR quantification of total RNA from the *lacZ* coding region in embryos, in either wt and Δ*homie* (in A), or wt and Δ*nhomie* (in B), normalized to control RNA from either *RP49* or endogenous *eve*, as indicated. **(A)** Line used in Fig 1B (H5 orientation). The reduction in expression is significant at the P < 0.01 level (one-tailed t-test assuming unequal variances). **(B)** Line used in Fig 1C (N5 orientation). The reduction in expression relative to *RP49* is not significant at the P < 0.05 level, but relative to *eve* it is significant at the P < 0.05 level (one-tailed t-test assuming unequal variances). Normalizing to *eve* expression may better control for variations in the developmental stages represented in the embryo collections that were the source of the RNA.
(DOCX)

S8 Fig Insertion of polyA-addition signals does not stop P-element promoter-initiated transcription. *eve-lacZ* and flanking P-element promoter-driven RNA expression in stage 5 embryos from *eve* pseudo-loci inserted at the 24B1 MiMIC site. Orientation of inserts are the same as in Fig 8. All loci have *homie* replaced with phage λ DNA (refer to maps). Positions and orientations of the RNA probes used are shown as black and red arrows in the map. Insertion site of polyA signals is shown in the maps of B and D. Affected *lacZ* stripes are labeled as 4, 5, and 6 (compare the intensity of stripes 4 and 6 to stripe 5 in the same embryos). **(A, B)** Same as in Fig 8A and 8B (ncRNA is driven by the P-element promoter), except that only the *α-tubulin* polyA signal is present here in B. **(C, D)** Same as in Fig 8A and 8B, except that here, the *NEE-PGFP* cassette used in Fig 7C is driving the ncRNA.
(DOCX)

S9 Fig There may be a slight reduction of P-element promoter-driven ncRNA downstream of polyA-addition signals. Quantification of total RNA (normalized to *RP49* RNA) from the indicated enhancer regions (shown as red bars) in the PΔ and PpA2Δ lines used in Fig 8. **(A)** Averages (with standard deviations) of 4 biological samples each are graphed. **(B)** Ratios of signals (average and standard deviation) from PΔ and PpA2Δ are graphed. **(C)** Quantification of *lacZ* RNA (normalized to *RP49* RNA). The increase in *lacZ* RNA in PpA2Δ relative to that in PΔ is significant at the P < 0.01 level (one-tailed t-test assuming unequal variances).
(DOCX)

S10 Fig Relevant end points and sequences used in this study. **(A)** End-point coordinates are given according to Flybase [74] (FB2021_02 release, R6.39). Numbers in parentheses are nt positions relative to the *eve* transcription start site. **(B)** List of sequences and primers used in this study.
(DOCX)

S11 Fig Data used for RT-qPCR graphs. (XLSX)
