# Supplementary figures and images for: An insulator blocks access to enhancers by an illegitimate promoter, preventing repression by transcriptional interference
Source: PLoS Genet. 2021 Apr 26;17(4):e1009536. doi: 10.1371/journal.pgen.1009536 (PMC8102011; doi:10.1371/journal.pgen.1009536)

###
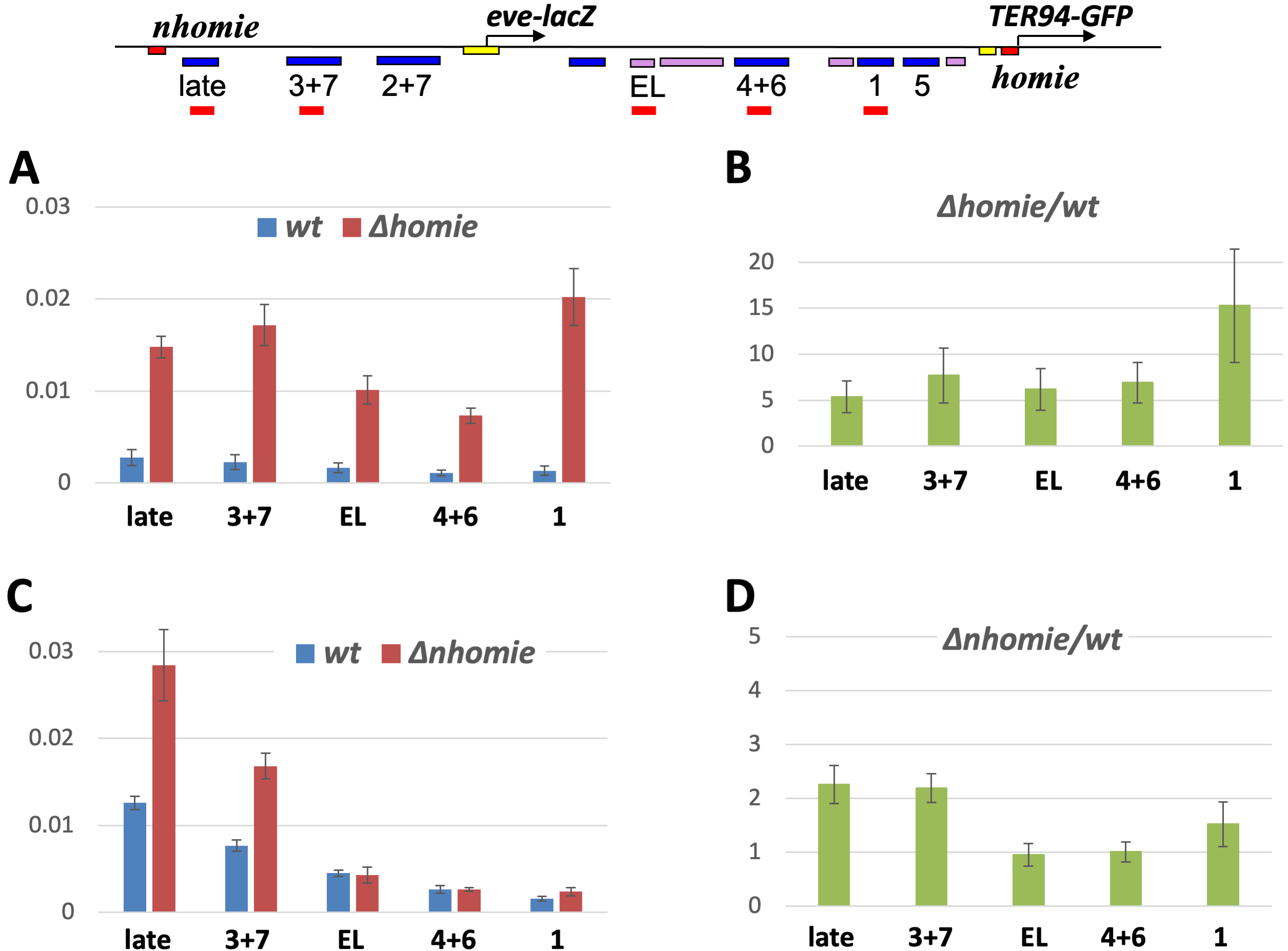


### S1 Fig.

Supplement: S1 Fig — (A, B) transgenes in the H5 orientation (see Fig 1A) at site 23C4. (C, D) transgenes in the N5 orientation (see Fig 1A) at 23C4. (A, C) RT-qPCR quantification of total RNA (normalized to RP49 RNA) from the indicated enhancer regions (probe locations shown as red bars below the map), in the wt and Δhomie transgenic lines (used in Fig 1D) in A, or in wt and Δnhomie (used in Fig 1E) in C. Averages with standard deviations of 3 biological samples each are graphed. (B, D) The ratios of average signals (with standard deviations) from wt and Δhomie in B, and wt and Δnhomie in D are graphed. Note the general trend toward a decrease in average signal moving away from the location of the 5’ P-element end (the right side in B and the left side in D). (DOCX) [file pgen.1009536.s001.docx]

###
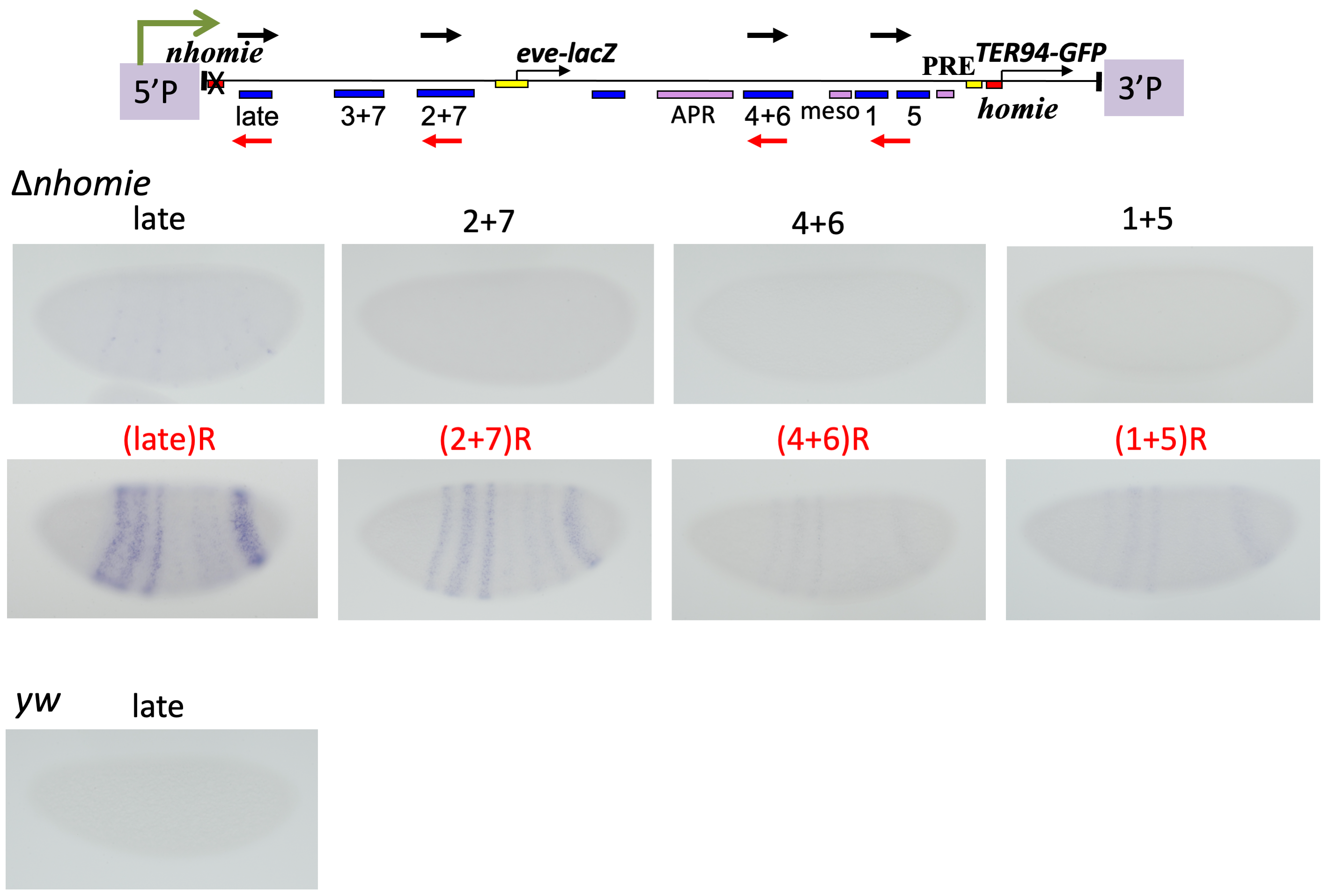


### S2 Fig.

Supplement: S2 Fig — Map: Transgene construct, showing location of the 5’ P-element end, where read-through transcripts are initiated. Images: RNA in situ hybridization to embryos at stage 5 carrying Δnhomie at 23C4 (used in Fig 1E). Probes are shown as either red arrows recognizing transcripts transcribed from left to right in the map, or black arrows recognizing transcripts transcribed from right to left. Labels use either black lettering corresponding to black arrows, or red lettering with R corresponding to red arrows. (DOCX) [file pgen.1009536.s002.docx]

###
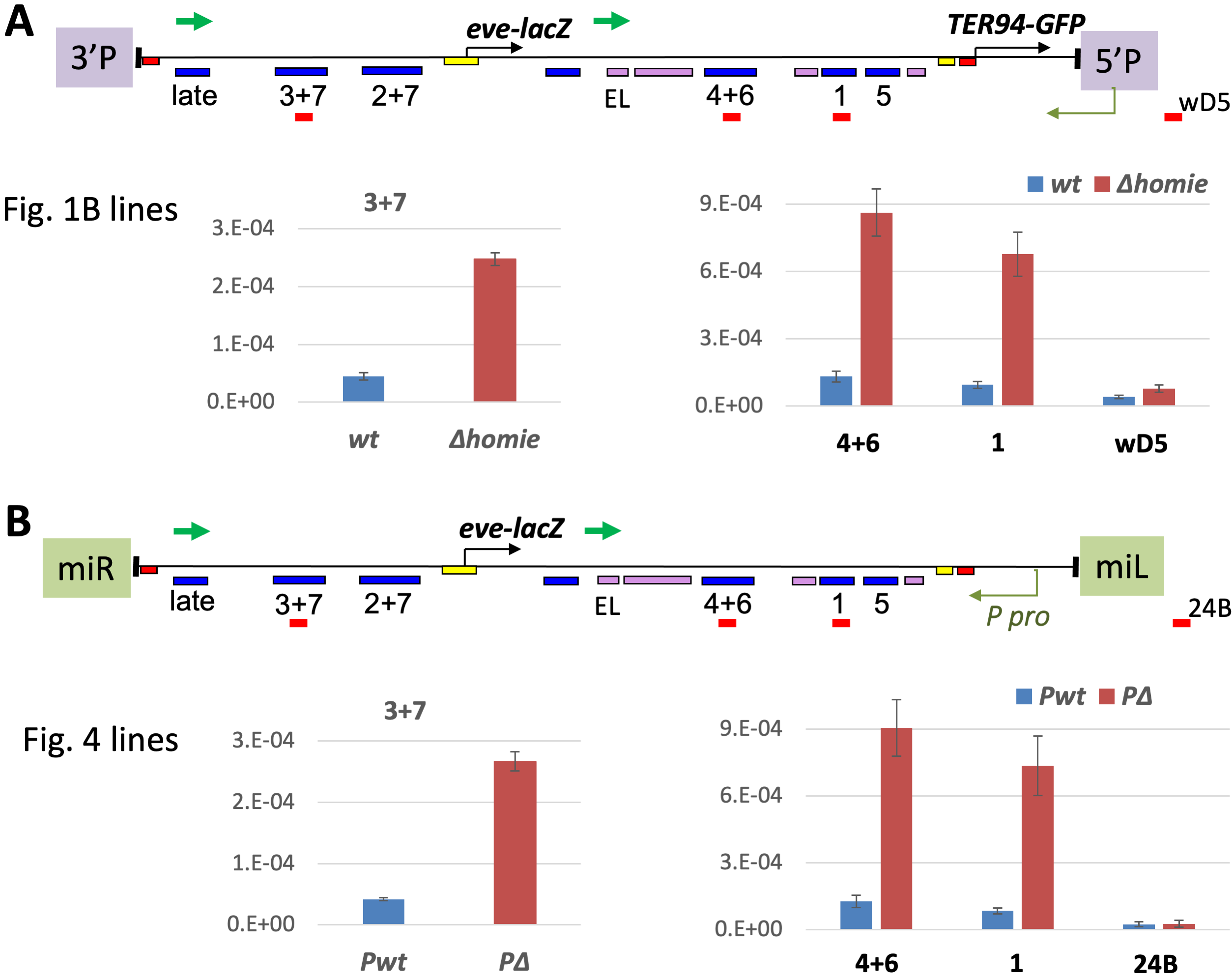


### S3 Fig.

Supplement: S3 Fig — cDNA primed in either the "late" or “EL” (green block arrows) enhancer regions were analyzed by qPCR using primers recognizing other enhancer regions (red bars below the map) (normalized to RP49, analyzed in parallel for each sample, using RP49-specific primers). (A) cDNA from wt and Δhomie transgenes at 74A2 (used in Fig 1B). Left graph: cDNA from "late" primer analyzed with “3+7” primer set. Right graph: cDNA from "EL" primer analyzed with “4+6”, “1”, and “wD5” primer sets. The "wD5" primer set was used to recognize cDNA representing the region upstream of the P-element promoter (a negative control). (B) Same as A, except that cDNA is from Pwt and PΔ transgenes (used in Fig 4A and 4B) at 24B1, and the “24B” primer set was used to recognize cDNA representing the region upstream of the P-element promoter (negative control). (DOCX) [file pgen.1009536.s003.docx]

###
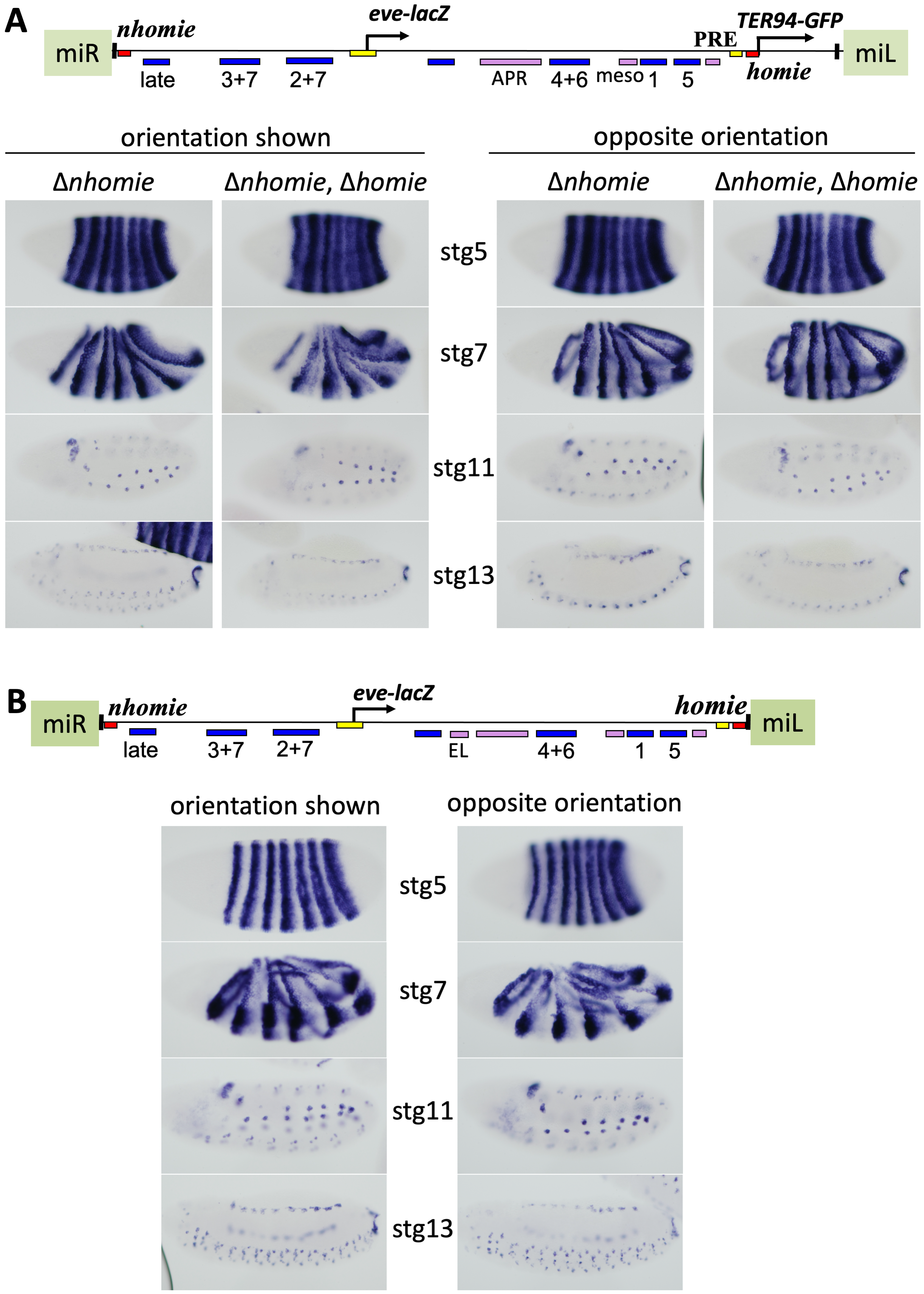


### S4 Fig.

Supplement: S4 Fig — (A) Top: Map of the eve pseudo-locus. miR and miL are Minos inverted repeats. Bottom: Expression of eve-lacZ from the pseudo-locus inserted into a MiMIC site at cytological location 24B1 detected by in situ hybridization. Both orientations of insertion are shown (left and right panels) at embryonic stages 5, 7, 11, 13, (as indicated), with either nhomie alone (Δnhomie), or both nhomie and homie (Δnhomie, Δhomie), deleted. (B) Top: Map of the eve pseudo-locus modified by removal of TER94-GFP. Bottom: Same as in A, using a line carrying this modified pseudo-locus. (DOCX) [file pgen.1009536.s004.docx]

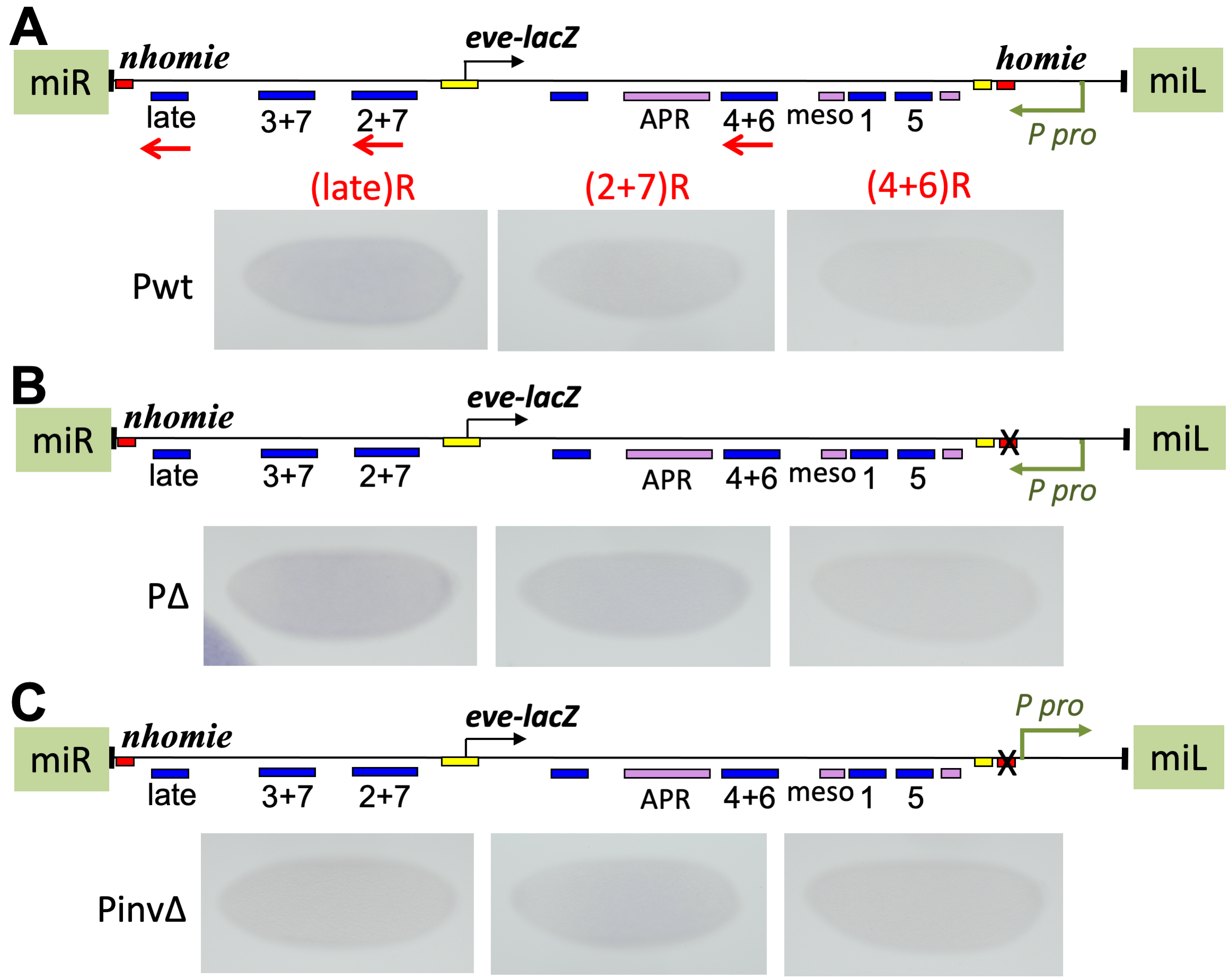


### S5 Fig.

Supplement: S5 Fig — Same as Fig 4, except probes recognizing the opposite strand are used. Positions of probes are shown as red arrows, and images are labeled with red lettering with R. (DOCX) [file pgen.1009536.s005.docx]

###
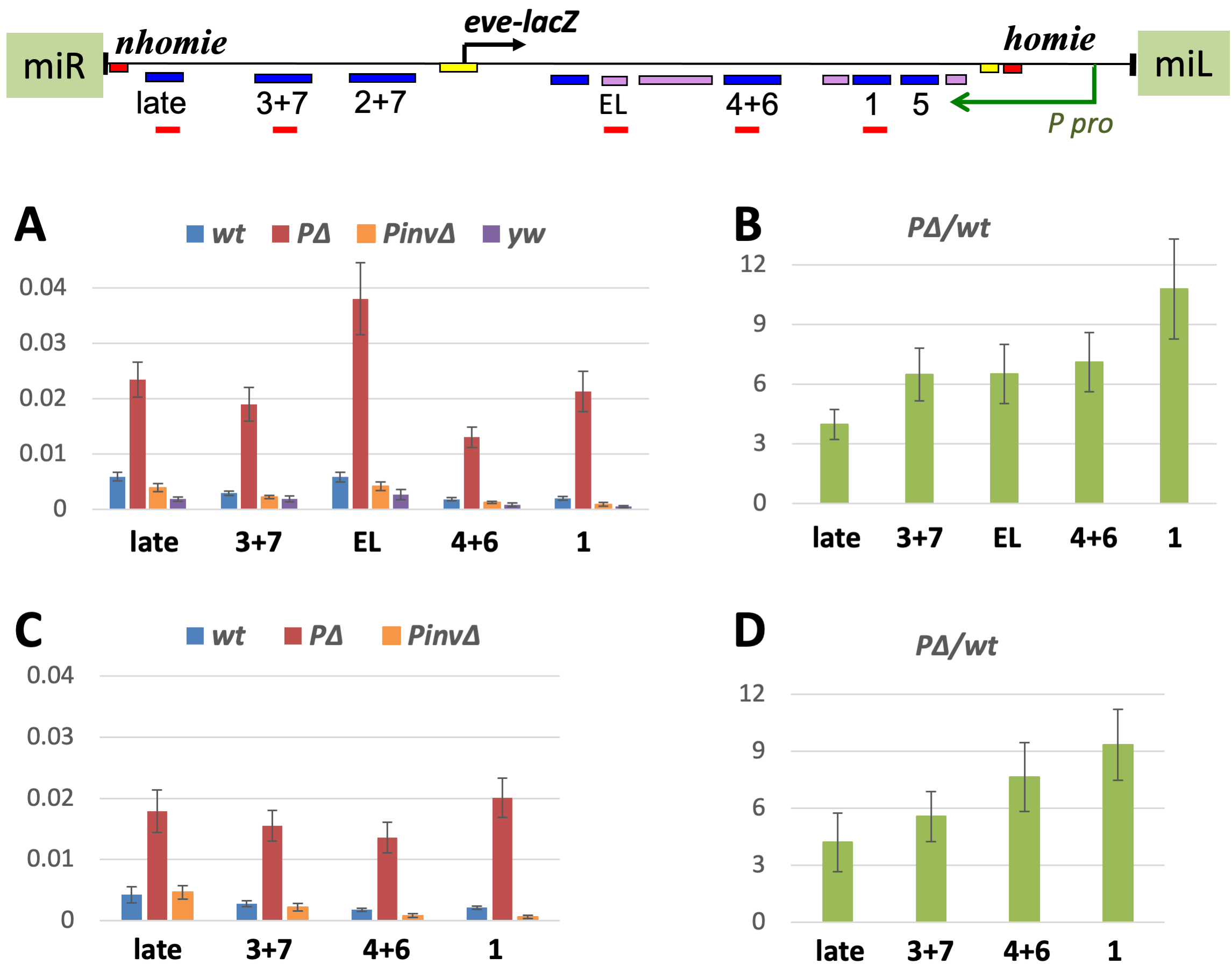


### S6 Fig.

Supplement: S6 Fig — Map: The wt transgene with locations of PCR products used for transcript detection shown as red bars under the map. (A) Loci used in Fig 4A (wt), Fig 4B (PΔ), Fig 4C (PinvΔ), and yw (without transgene; signals come only from endogenous eve) were subjected to RT-qPCR quantification of total RNA (normalized to RP49 RNA) from the indicated enhancer regions. Averages (with standard deviations) of 4 biological samples each are graphed. Note that transcript levels are strongly increased throughout the locus in PΔ, but not in wt or PinvΔ. (B) The ratios of average signals (with standard deviations) from PΔ and wt are graphed. Note the general trend toward a decrease in the relative PΔ signal moving away from the location of the P-element promoter. (C) Similar to A, except that the set of wt, PΔ, and PinvΔ transgenes are inserted in the opposite orientation at the same MiMIC site, and averages (with standard deviations) of 5 biological samples each are graphed. (D) The ratios of average signals (with standard deviations) from PΔ and wt in C are graphed. (DOCX) [file pgen.1009536.s006.docx]

###
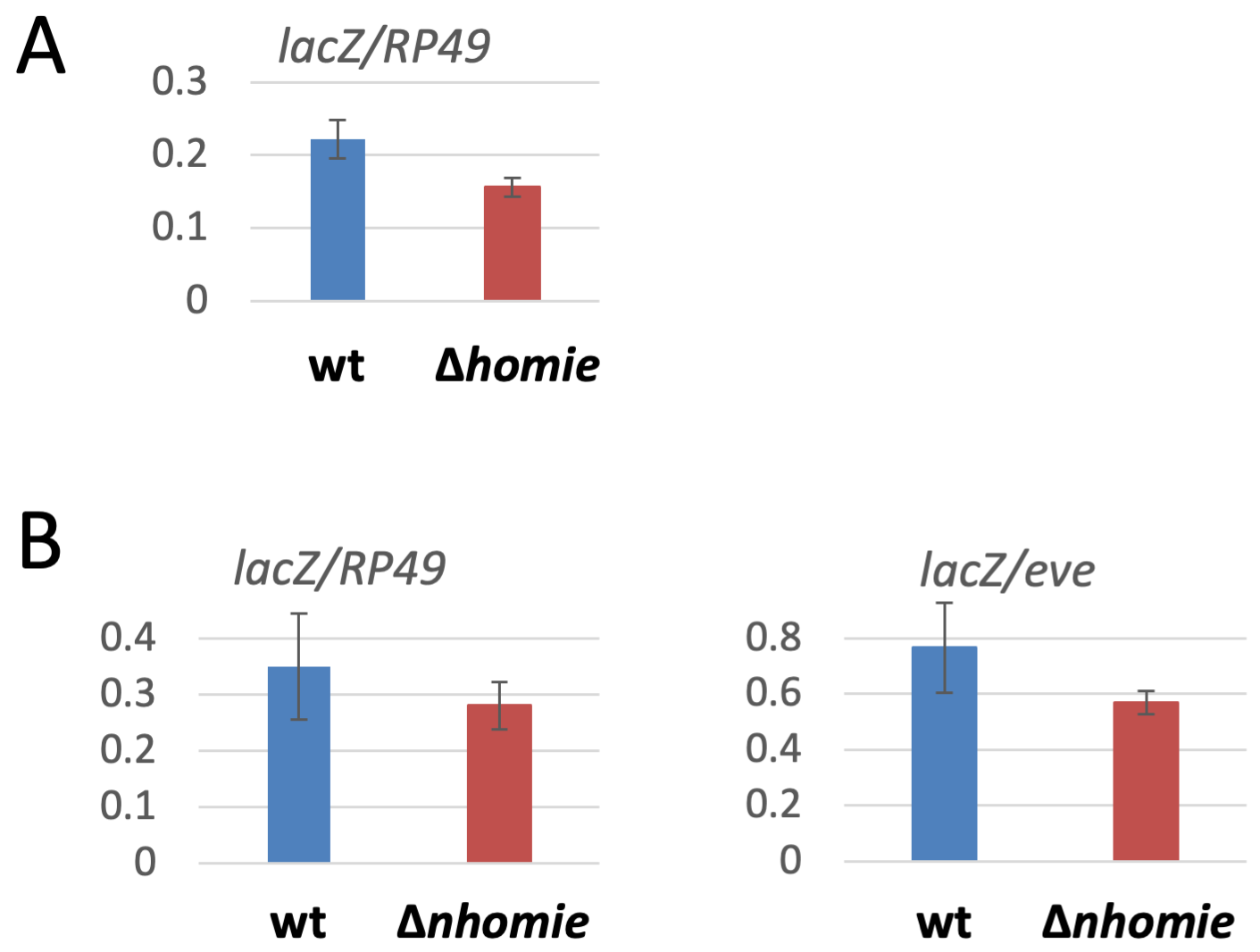


### S7 Fig.

Supplement: S7 Fig — RT-qPCR quantification of total RNA from the lacZ coding region in embryos, in either wt and Δhomie (in A), or wt and Δnhomie (in B), normalized to control RNA from either RP49 or endogenous eve, as indicated. (A) Line used in Fig 1B (H5 orientation). The reduction in expression is significant at the P < 0.01 level (one-tailed t-test assuming unequal variances). (B) Line used in Fig 1C (N5 orientation). The reduction in expression relative to RP49 is not significant at the P < 0.05 level, but relative to eve it is significant at the P < 0.05 level (one-tailed t-test assuming unequal variances). Normalizing to eve expression may better control for variations in the developmental stages represented in the embryo collections that were the source of the RNA. (DOCX) [file pgen.1009536.s007.docx]

###
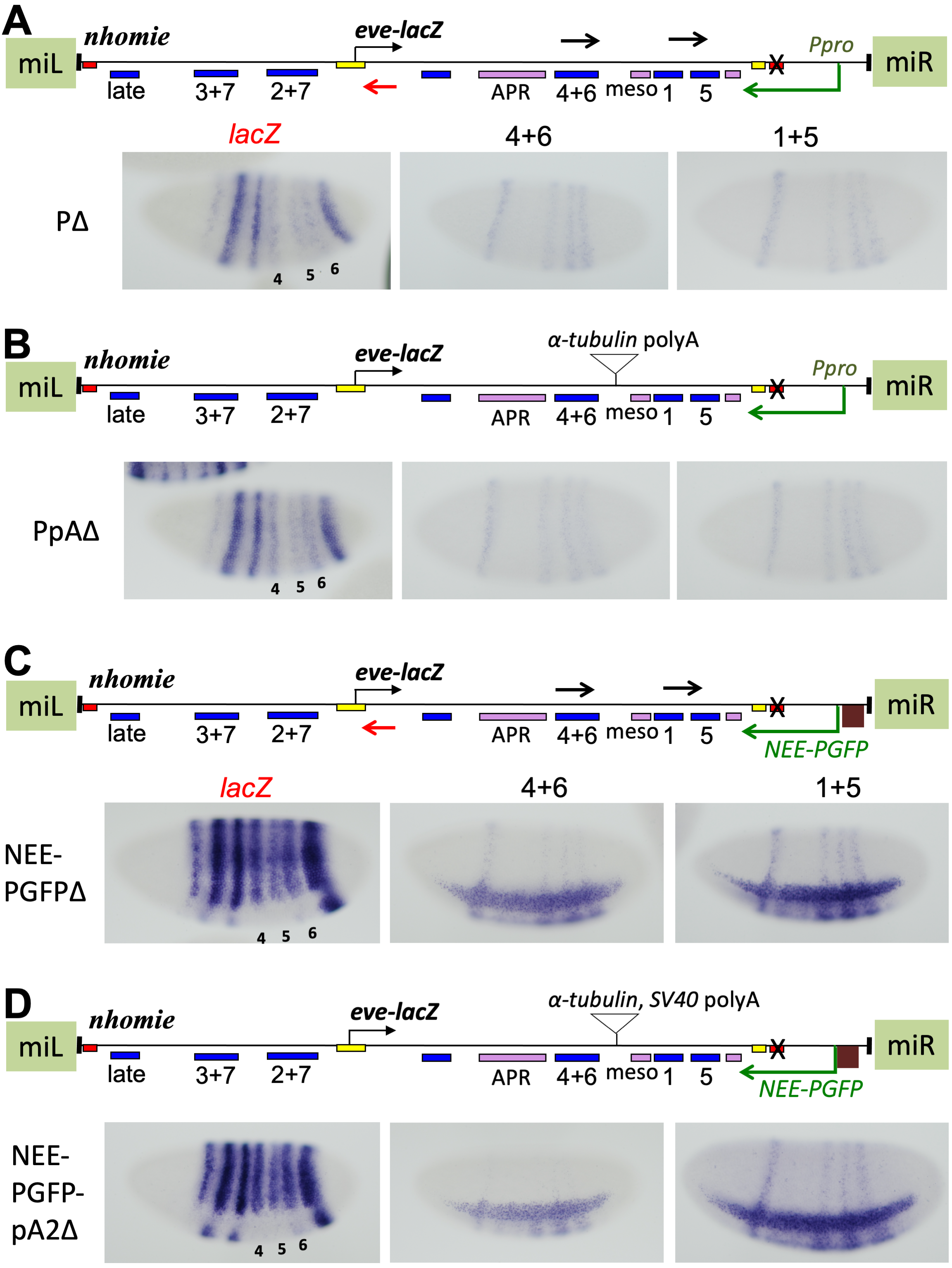


### S8 Fig.

Supplement: S8 Fig — eve-lacZ and flanking P-element promoter-driven RNA expression in stage 5 embryos from eve pseudo-loci inserted at the 24B1 MiMIC site. Orientation of inserts are the same as in Fig 8. All loci have homie replaced with phage λ DNA (refer to maps). Positions and orientations of the RNA probes used are shown as black and red arrows in the map. Insertion site of polyA signals is shown in the maps of B and D. Affected lacZ stripes are labeled as 4, 5, and 6 (compare the intensity of stripes 4 and 6 to stripe 5 in the same embryos). (A, B) Same as in Fig 8A and 8B (ncRNA is driven by the P-element promoter), except that only the α-tubulin polyA signal is present here in B. (C, D) Same as in Fig 8A and 8B, except that here, the NEE-PGFP cassette used in Fig 7C is driving the ncRNA. (DOCX) [file pgen.1009536.s008.docx]

###
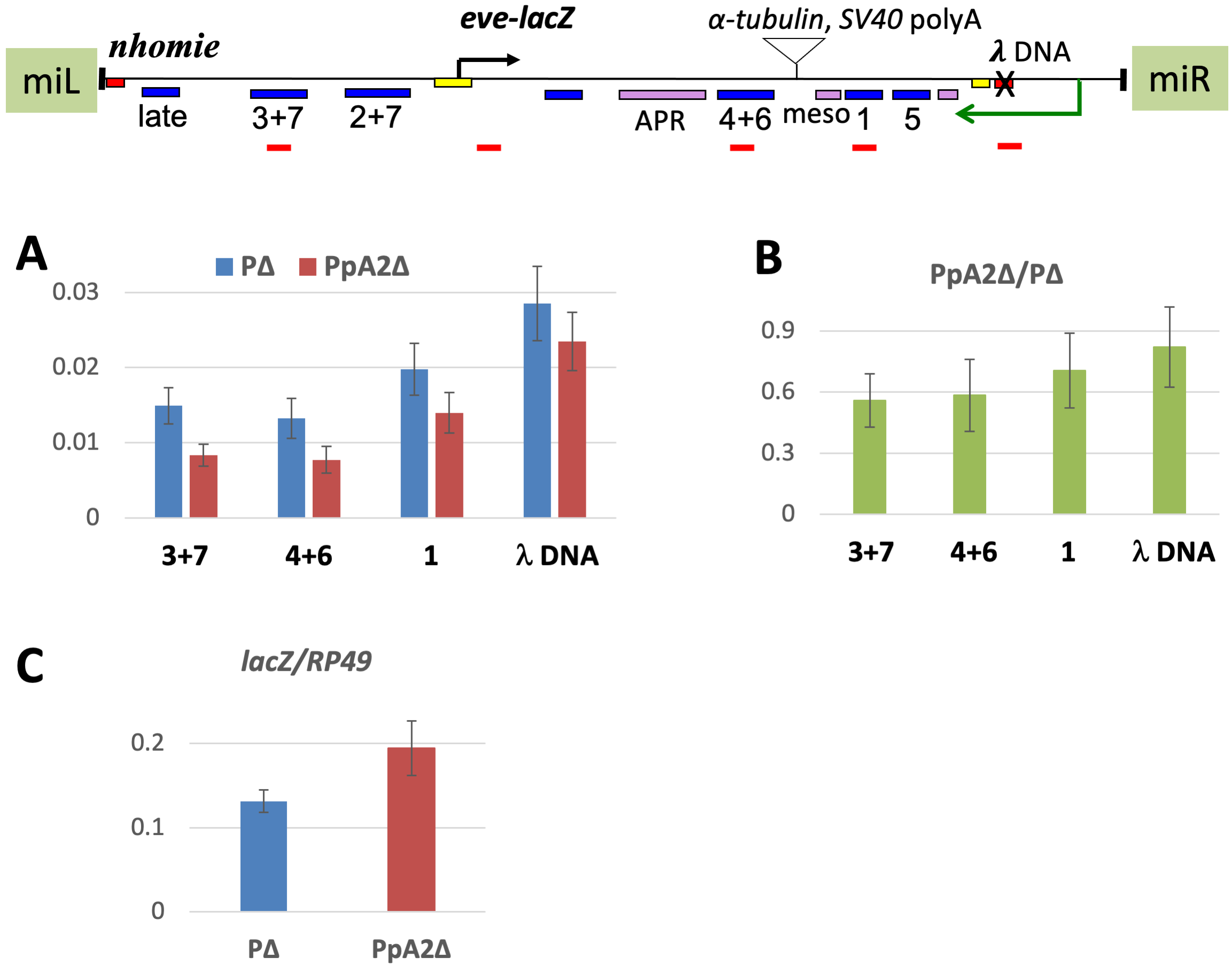


### S9 Fig.

Supplement: S9 Fig — Quantification of total RNA (normalized to RP49 RNA) from the indicated enhancer regions (shown as red bars) in the PΔ and PpA2Δ lines used in Fig 8. (A) Averages (with standard deviations) of 4 biological samples each are graphed. (B) Ratios of signals (average and standard deviation) from PΔ and PpA2Δ are graphed. (C) Quantification of lacZ RNA (normalized to RP49 RNA). The increase in lacZ RNA in PpA2Δ relative to that in PΔ is significant at the P < 0.01 level (one-tailed t-test assuming unequal variances). (DOCX) [file pgen.1009536.s009.docx]
