# Supplementary material for: An insulator blocks access to enhancers by an illegitimate promoter, preventing repression by transcriptional interference
Source: PLoS Genet. 2021 Apr 26;17(4):e1009536. doi: 10.1371/journal.pgen.1009536 (PMC8102011; doi:10.1371/journal.pgen.1009536)
Supplement: S10 Fig — (A) End-point coordinates are given according to Flybase [74] (FB2021_02 release, R6.39). Numbers in parentheses are nt positions relative to the eve transcription start site. (B) List of sequences and primers used in this study. (DOCX) [file pgen.1009536.s010.docx]

### S10 Fig.

**
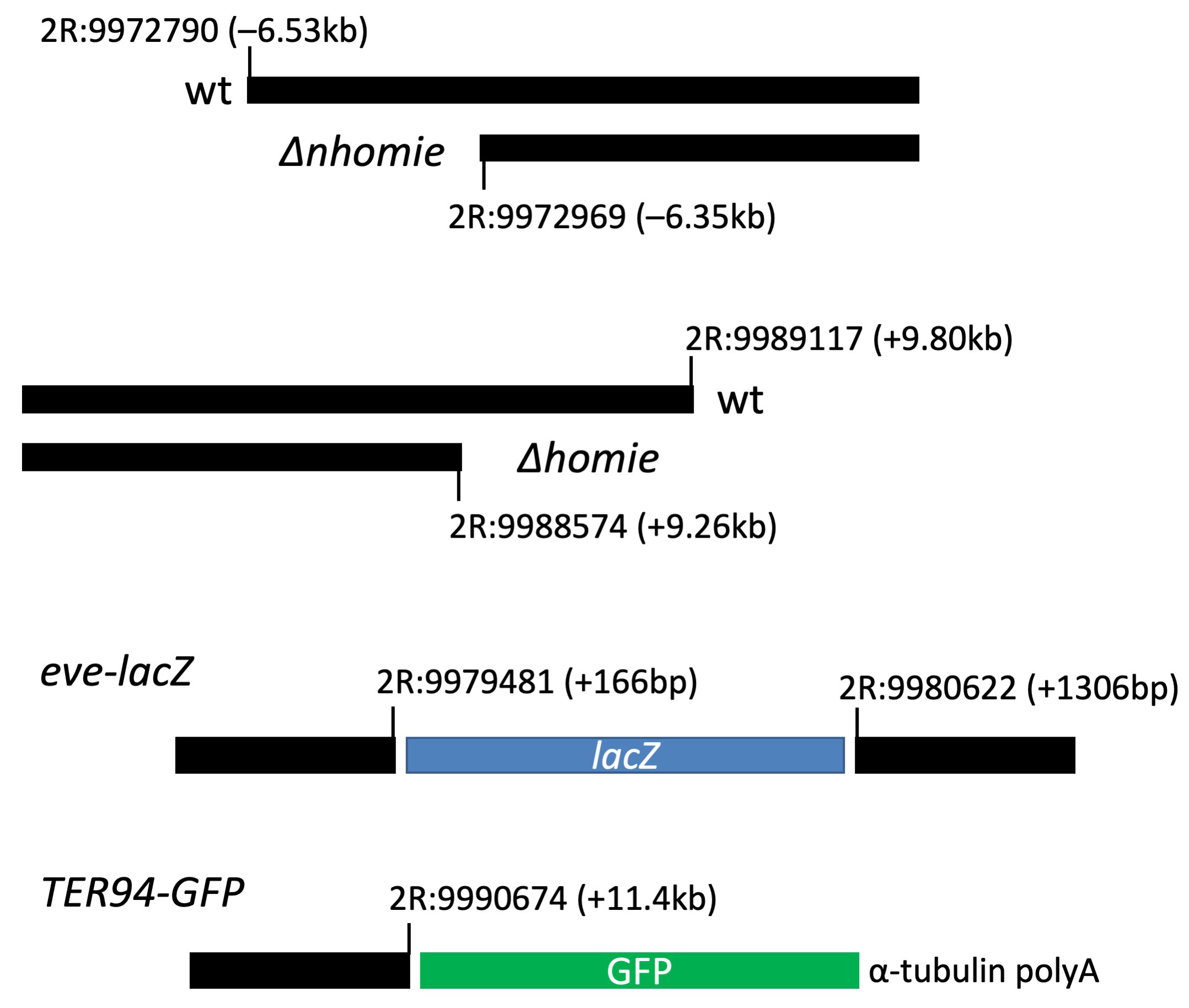
(A)** End-point coordinates are given according to Flybase [1] (FB2021_02 release, R6.39). Numbers in parentheses are nt positions relative to the *eve* transcription start site.

**(B)** List of sequences and primers used in this study.

**Coordinates of wt and ∆ for *homie* and *nhomie***

**Flybase [1]: FB2021_02 release (R6.39)** (*eve* +1: 2R:9979319)

wt *nhomie* 5' end: 2R:9972790

∆*nhomie* 5' end: 2R:9972969

wt *homie* 3' end: 2R:9989117

∆*homie* 3' end: 2R:9988574

**Coordinates of *eve-lacZ* and *TER94-GFP***

3' end of the *eve* region (fused to *lacZ* 5' end): 2R:9979481

(fused to *lacZ* 3' end) 5' end of the *eve* 3' UTR: 2R:9980622

3' end of *TER94* region (fused to *EGFP* 5' end): 2R:9990674

**Promoters and enhancers used**

P-element promoter

CATGATGAAATAACATAAGGTGGTCCCGTCGATAGCCGAAGCTTACCGAAGTATACACTTAAATTCAGTGCACGTTTGCTTGTTGAGAGGAAAGGTTGTGTGCGGACGAATTTTTTTTTGAAAACATTAACCCTTACGTGGAATAAAAAAAAATGAAATATTGCAAATTTTGCTGCAAAGCTGTGACTGGAGTAAAATTAATTCACGTGCCGAAGTGTGCTATTAAGAGAAAATTGTGGGAGCAGAGCCTTGGGTGCAGCCTTGGTGAAAACTCCCAAATTTGTGATACCCACTTTAAAGATTCGCAGTGGAAGGCTGCACCTGCAAAAGGTCAGACATTTAAAAGGAGGCGACTCAACGCAGATGCCGTACCTAGTAAAGTGATAGAGCCTGAACCAGAAAAGATAAAAGAAGGCTATACCAGTGGGAGTACACAAACAGAGTAAGTTTGAATAGTAAAAAAAATCATTTATGTAAACAATAACGTGACTGTGCGTTAGGTCCTGTTCATTGTTTAATGAAAATAAGAGCTTGAGGGAAAAAATTCGTACTTTGGAGTACGAAATGCGTCGTTTAGAGCAGCAGC

*hsp70* core promoter (sequence downstream of **ATG** is included in *NEE-hspGFP*, but not in hsp∆)

CGCCGGAGTATAAATAGAGGCGCTTCGTCTACGGAGCGACAATTCAATTCAAACAAGCAAAGTGAACACGTCGCTAAGCGAAAGCTAAGCAAATAAACAAGCGCAGCTGAACAAGCTAAACAATCTGCAGTAAAGTGCAAGTTAAAGTGAATCAATTAAAAGTAACCAGCAACCAAGTAAATCAACTGCAACTACTGAAATCTGCCAAGAAGTAATTATTGAATACAAGAAGAGAACTCTGAATACTTTCAACAAGTTACCGAGAAAGAAGAACTCACACACA**ATG**CCTGCTATTGGAATCGC

*rhomboid NEE*

CCTTGGGCAGGATGGAAAAATGGGAAAACATGCGGTGGGAAAAACACACATCGCGAAACATTTGGCGCAACTTGCGGAAGACAAGTGCGGCTGCAACAAAAAGTCGCGAAACGAAACTCTGGGAAGCGGAAAAAGGACACCTTGCTGTGCGGCGGGAAGCGCAAGTGGCGGGCGGAATTTCCTGATTCGCGATGCCATGAGGCACTCGCATATGTTGAGCACATGTTTTGGGGGAAATTCCCGGGCGACGGGCCAGGAATCAACGTCCTGTCCTGCGTGGGAAAAGCCCACGTCCTACCCACGCCCACTCGGTTACCT

***homie* replacement by λ DNA and known insulators**

λ DNA

GGATGCCAGTGATAAGTGGAATGCCATGTGGGCTGTCAAAATTGAGCAGACCAAAGACGGCAAACATTATGTCGCGGGTATTGGCCTCAGCATGGAGGACACGGAGGAAGGCAAACTGAGCCAGTTTCTGGTTGCCGCCAATCGTATCGCATTTATTGACCCGGCAAACGGGAATGAAACGCCGATGTTTGTGGCGCAGGGCAACCAGATATTCATGAACGACGTGTTCCTGAAGCGCCTGACGGCCCCCACCATTACCAGCGGCGGCAATCCTCCGGCCTTTTCCCTGACACCGGACGGAAAGCTGACCGCTAAAAATGCGGATATCAGTGGCAGTGTGAATGCGAACTCCGGGACGCTCAGTAATGTGACGATAGCTGAAAACTGTACGATAAACGGTACGCTGAGGGCGGAAAAAATCGTCGGGGACATTGTAAAGGCGGCGAGCGCGGCTTTTCCGCGCCAGCGTGAAAGCAGTGTGGACTGGCCGTCAGGTACCCG

Fab-7

GTGGCAAAAGCTGGCAAAGCAGCAAAAATCGTAAAAAAGAAAATTGCATTTCCCCAAAGCAGCGAAACTTGCGCAGGACTTTTGAGATTCTATTAAATTCTAACAAGATTTCAAGCTGTGTGGCGGGGGAAAGAGGAAGAGAGCGGAAAGTGCAGCGCCCAATAAGCAAATGGCAGCTGTCACGGGGAAGCACAGAGAGTGCAGAAAGGGGAAAAAACATTGGGGCATATCAACGCGCCAAAAAGAAAAACAAAAAGAGCGAGGTAGAATGTCGCTCAAAGAGCGACACGTGAACAGGTGCAGTAGTAAATATAAGCAAAGAGAGTTGGAAAGAGTATTGGCTAAGAGCGTCCGCTCACTAACACATAGATAAATTAAGAGAGACGTGATAAGAGAACCGCACGCACACCACCGCAAAATCCAATTGGAAGAGAGCGACTGCTTGAGTGTATTGGTTAGCAAGAGAGCGGCTAGGTTTGATGGTTTGATTGGAATTCAGTTGCCGTTCGAAATATTTTTGATAAAATATAAAAAATAATTCAGAGGCTGAGGCAAGTCTAAAAACAATGCTTTGCCTAAGAATTCGTAGCTTTTATAATTTTTTTTTAAATTTTGAAATTAGCATTTTATTTTTTAAATGATTCTCCAATTAAGCCAACTGGTTTCCAAATCTAGCGGCGAACTTCACCTTTTGGTTTGCGTACCGACTAAGTCCGAGCAGTGCTGCGCATCCTTTTTGAGCCTTAGTATACCCATCTCGCTCTTAGCCACCCCTAAATACCGTTACTTACCCTGGGCAACTTCCTTCGTCCGTCGGCCTTTGTTTCTGCATTTTTTTTGTTTTTGTCTGGGCGACGACGCAGTCGCAGAAAGTCCCTCGAAATTCCTCCGCTCCCTCGCTCGCTCACAATCCTGT

Fab-8

AATTCAAATTTATAATGAAAGTTATTGTTATAAAAATGTACGTCTACATATGCTTCATGTACATATATGTATGTCTTATAATTTATGAAAAATTATATCAAACGAAATAATATTAACGAAAACATTTTTTATATATGCAGACATCTTCCGTTCATCCGTTTCAATAATATAAGGAAAGATTTTGAAAAGAGATAATGACATGGCACAATCAAGTTAATGTTGGAAATGTAATTATATCTTAACCTTTATTTAAATGTGAGTTGTTAGGATTTTTGAATATAAGAACTGGTGTCTTAAATACACTTATTGTCTGTTCAATCGAACCATTGAAAGTTATCGAACATTTTTTACGCGACATGTCCACTGTCGGAGAGCGACATCTTGTGTTGGTGCTTTCCTTCCTAGTTCTACATTACCAAGGCCAGGTGGCGCTGCAAGGCGGGAATGTGGAAATTTAAAGTACTCTTTCTCTCTTCGTACTTCGAAGCAGAGAATGGAACTCTTCGCTTGCTCACCAACACAAATGCCTTGCACAGCTTGCCACTGTGTGAGTGAGTCTTATTCGAATGTGTTTGTGCTGGTTGGCGTTGACGTCGATTTCGGATTTCTGCTTTCTGAGCAGAAAAATGCCGTAA

*gypsy*

GTATGGCAAGAAAAGGTATGCAATATAATAATCTTTTATTGGGTATGCAACGAAAATTTGTTTCGTCAAAGTATGCAATATTTTTTATTAAAAGAGGGTATGCAATGTATTTTATTAAAAACGGGTATGCAATACAATAATCTTTTATTGGGTATGCAACGAAAATTTGTTTCGTCAAAGTATGCAATATTTTTTATTAAAAGAGGGTATGCAATGTATTTTATTAAAAACGGGTATGCAATAAAAAATTATTTGGTTTCTCTAAAAAGTATGCAGCACTTATTTTTTGATAAGGTATGCGACAAAATTTTACTTTGCCGAAAATATGCAATGTTTTTGCGAATAAATT

**Genomic regions used for *in situ* hybridization probes**

1 + 5

AGGCCTAATCACTTCCCTGAAATGCATAATTGTGCCGCGGCTTTTGATACGCTCCTGGCGGAGAGGGAGATGAGGAAAGGATGCACGGGAACCGCAGCCAAGTGGCAGTCGAGATTGGCAAATCCGCCAGCGGACAATGCCCAGAGAATGGGCAACAAGTAGCGGCGAATTAGCAATCCTATCATGCTTTTATGGCCGGCCAACTCTTGCCCGCGCATCTCAGTTCATCCGAAGCGGGACCAGGTCCAGGTTCAAGTCGAGGTCCAGTACCCCTGCTATCCCGTCAACCCCTTTAGGGCGATAATCCTTCTAAATGTTTGCATTAATTTCGAGGCGTGGACGGATTAGGGCGTGCTGGCTGGGCGGAACCCGCAGCAGAAACCGCCGAGGACACTGCACCGACTGACCTGCAGCCTACAGATCTCTGATCTTCGATCTCTAATCCTTTCGCATTTGCAACTGACTTCTGCACTGGGTCCGCCCCTAATCCTTCCGCCGAGAAGGCGGCAGAGTCGCGAGGTACTGGCCCGGGGTAATGGGATTATCTGCGATTACCCCAGATGATCCGCAGAAAGTCAATCTGGTTCAGGGGCTAATTGTCAGCGAAGTCAACTAAATCCAATCCTTTCGCGCCCCCTTCTGTTTATTTGTTTGTTTTCGTTTGTTTTGAGAATTTCTGGCAATTAAGTTGCCCGTTTTGATGCGCGGGGGCGGGTGCATCAAATCCTTTCGGCATACCTGTCCTGCACAAATGCTGAATTCCGCATCCCATGGATACCCAGATATTCAGATATCCCAAGGCCGCAAAGTCAACAAGTCGGCAGCAAATTTCCCTTTGTCCGGCGATGTGTTTTTTTTTTAGCCATAACTCGCTGCATTGTTTGGGCCAAGTTTTTCTTCTGCCAAATTGCGGAGATGATGCGGGGATTATGCGCTGATTGCGTGCAATTATGGACATCCTGCGAGGCCCCGAGGAACTTCCTGCTAAATCCTTTCATCCGCCTACAGAACCCCTTTGTGTCCCGTTCGCCGGGAGTCCTTGACGGGTCCTTCGACTATTCGCTTACAGCAGCTTGCGTAAAATTTCATAACCCTACGAGCGGCTCTTCCGCGGAATCCCTGGCATTATCCTTTTTACCTCTTGCCAATCCGTTGGCTAAAAAACGGCTTCGACTTCCGCGTAACTGCTGGACAACAAAGACAAAAAACGGCGAAAGGACGGCGATTTCCAGGTAGCATTGCGAATTCCGTCAAACTAAAGGACCGGTTATATAACGGGTTTATATGGCCAGAATCTCTGCATCTCCACGACCGCCAGAAGCTGCGTAAAACTGCAGGCTCTGTTTTGATTTCTGCAACTTCAGTTAATTGCCCGGGATGGCCAGCAATTGCCGGCAATTATAAAACAGCGCAGATGTGACTCAGCTTCCATATCTAACTCTATATCTCATGCCGAAAATCTAGGGTGGGGAGCGGAGGGGCGGGGTGCGTGGGTGACTTGCCTGCCAGGGAAAGGGGGCGGGGGTTCAGCGGGTGATAAATGTGCGTGATTTGGAATGAATGCGCATCGATTAAAACCGCAGGGCAATCAATTTAGCGCCTTTTACGCCAAATTGGCTCGTACACAACCAATTAATGTCAGCGGGTGAACTGACACCATCGCCCACCACCGCATCCCCCTTTCCCCTGTTGGCCATCCACCCCCGAAAAACAATTACAACAACGAAGACAAGCAGAGGGACTGCTGCAGATTCCGCTCAATAAACCTCCAATAAAGCGAATCCAGCGTGAGGCGTCGACGTC

4 + 6

TCGCGACAATCAAACTCGTGTTTAGCCAACAGTCGCAGCATTTCCATACCATGGGGGTGGTCTCTGCTGGGTGTTGCGAATGCGACGCCAATTGGTCAGGGTTCACTGTAAATGGCCCAGCCAAATGGGAAGCGGCAGTTGAGGAGCGCCCGAATCAATTGCCCTGATGGATGCTGCGGCTGTCCAAAGTTGCAGCTTTTCGGGTCACTCCGCGCTGGGGCTGGAGGGCTATAAATCCGCAGGCCAGATAATGAAACTAGAATGATTGAGGCAATCACTGGTGTGGCCAGCAGTCTGCTGGCGGAGTTTCCTACGCTCTGCGCATGTCCTGGATACACACAGCTCGGCGCACATAGCGATAGATACAGATGCAGATACAGATACAGAGGCAGATAGATACGTATGCGGATACATAGAGCACGTTACATTATGTGGCGAAGGACTGGCGATTACCGATTCCGCAGTTCAGGACCTCTGGATTTGCGATCCTGCGCACGACGTGTCAACTTTATTGCGGTTTGACTTTGCCGCGGCCCCTCGAAACTCACAAACGTATCCTGGAACCCAGGATCCCTGGGCTCTGGGCTCTGGACTATCCGCCGACCCTCCATATCCATGATTTACAATTCTCGTTTTTTTCGCGTTATTTTTTTAGGGGCTTTAATGACCGTCGTAAAGCCGCAGGAGGACCAGGACCAGGACTCTGCTCACATTTCGCGCACTGATTCTAAAAAATGAAATCATTTTTTCTTGAATTTCACGGCGCGCCTCGAGCAGGACTCTTTGTTCTCGGCCAGGCAATTGTCCTTTTTTGCGCTCAGCTCTCAGTTTTTTCGTCCAGCGGGCATTACCTACACGGCGTTTTATGGCGGAGATGATATTCGCCTGGGATCGGTTCCGTTTTTTAGGCCATAAAAATTAGGCGGCATAAAAAAACTGCATTGGAATTCTAGTTCTAGTTTCAAGTTTTTAGGTTTCCAGGTTTCTGCCAGCCCGCCTAGATTCGCATTTCGCGGAATTCGGAAGCGGAACAGAATGCCAGAATGGTCAGAATCCTGGCTGACCTTGCCTTTTGGCCAGGGGCCGTAAAAAAATTGACTCGCTGCGGTGCGCGGAATATTTTTTAAATCTGACTTTCCAACAATCTCTGATCTGGGTTCGAA

2 + 7

TCGAGAATTGAACCTTTCCCGGGGCAAGAAGGCTTGCATGTGGGCCTTTTCCAGGTCGGCCAGTAGGTAGAGTTGTTGCGATGCGGCTATGCCGGGCGAGTTAATGCCAATGCAAATTGCGGGCGCAATATAACCCAATAATTTGAAGTAACTGGCAGGAGCGAGGTATCCTTCCTGGTTACCCGGTACTGCATAACAATGGAACCCGAACCGTAACTGGGACAGATCGAAAAGCTGGCCTGGTTTCTCGCTGTGTGTGCCGTGTTAATCCGTTTGCCATCAGCGAGATTATTAGTCAATTGCAGTTGCAGCGTTTCGCTTTCGTCCTCGTTTCACTTTCGAGTTAGACTTTATTGCAGCATCTTGAACAATCGTCGCAGTTTGGTAACACGCTGTGCCATACTTTCATTTAGACGGAATCGAGGGACCCTGGACTATAATCGCACAACGAGACCGGGTTGCGAAGTCAGGGCATTCCGCCGATCTAGCCATCGCCATCTTCTGCGGGCGTTTGTTTGTTTGTTTGCTGGGATTAGCCAAGGGCTTGACTTGGAATCCAATCCCGATCCCTAGCCCGATCCCAATCCCAATCCCAATCCCTTGTCCTTTTCATTAGAAAGTCATAAAAACACATAATAATGATGTCGAAGGGATTAGGGGCGCGCAGGTCCAGGCAACGCAATTAACGGACTAGCGAACTGGGTTATTTTTTTGCGCCGACTTAGCCCTGATCCGCGAGCTTAACCCGTTTTGAGCCGGGCAGCAGGTAGTTGTGGGTGGACCCCACGATTTTTTTGGCCAAACCTCCAAGCTAACTTGCGCAAGTGGCAAGTGGCCGGTTTGCTGGCCCAAAAGAGGAGGCACTATCCCGGTCCTGGTACAGTTGGTACGCTGGGAATGATTATATCATCATAATAAATGTTTTGCCCAACGAAACCGAAAACTTTTCAAATTAAGTCCCGGCAACTGGGTTCCCATTTTCCATTTTCCATGTTCTGCGGGCAGGGGCGGCCATTATCTCGCTACAGCAGTTCCCAAATGGTTATGGCTGGACACCCCTGCCGCCGCTCCAACGGGGTGGATGAAGCCCCCAAAACCCGAAAGTCATGGCAGCCATGGCAGTGTGGGGCTGTTAAACGTGCGGCATAATATTAAGACTTCATAAAAGCGCAAATAATTCGCTGGCAGGCGATCGATAATACATACATACAAATATATAGTGGGATACACACACTCTCTGCCGGCAAACACACACCACCCGACCCGACTGAGCGGCATAATGCCATATCATTCTTGATGAAGCCGATAAAATCCCATTATTAAGGGGGCCCGCCCGTCCCGCTCGCTCCTGCGGAGCAACCGCCTGCGGGCGGGCGAGACAAAAGATTCGCTCATCCGCTATGAATACCAAATCGGAACTCTCTCTCTCTCCAGCTCGGGAGTGCCATGGCCAGCATGGCCAGGACCTCCTCATGGTCCTGCCGAGCAGAGAACGCGGCTCCATCCCGCTGCTCCGGGTCCTGCTCCTCCGCTTTGTCCCGCCTCGTTATCGCCGCTCAGCACCGAGAGCACAGCAGCGCATCCACTCTCAGCACCGCACGATTAGCACCGTTCCGCTCAGGCTGTCCCGCTCGCACCTGCCTGGGTCGCTGCGATTGGCCGCTCCCAGCGACGGCGGCCATTTGCCTGCAG

late

ACATATGTAAATAAAAATTGGGTTCACTGTATATAAAATAGAGTATAAATTATTCATCTTATCTAACAAATATTAGAAATAATCAAAACACCTTATAAGTTTATCGAATTTACGAATTTACGAATTTATTTCTACATATAAATGTTGACCGCCATTTGGACTCCACTTAACCGCTTAATTTGCTTAAGTAACATAAGTAACTTTTAAGATTAATAGTATTGTATTTTGAATGGTAAAAGTCCATCCTAATCTATTGAAAAAATCCGAAGGAACCAAAGTCATTCAAAGTTGATTCAATTCACCCCCACCCTGCGCACTCAGTGCTCAATTTCTGCTCGCAGCAAGGTCTGCTGAGTAAAAAATTAGCACATCCTCAGCGCAGATAAACGCATCCTTAAAACACCCCCTGGCCATCCCACAAGCTAGCCATCCATCCATCCGTTCATCCTGCATCCGCATCCCTTCGCACAGAGAGCGGCCCAATTAGTGCTGCAGTAGGCATTTCGAATCCGTACCCACAAATCGGCTAATCCGCTGCCACTGCCTGCCGGCCCAGAGTCCAAAAGTCCTGTGTGCAATGTCCAGATTTCCGAGTCGCAGAGTCTAAATGTTGACAAATACAACACCTACGATACAAATAAAATTGCTACAGAAGTACCACCTAAAAACGGGCACAACCGGAACGACTTAATTTTCTCAAATTTACAGAAACAGACGATGACATAGGAATGGGAATGGTGATGGTGATGGAAACACAGGAAGATATCTCGACGCCGCCACTGAAGCTGTGGGTTTGCTCCTGCCGAGCGAATCCAACGCGAGTAGGGTCCCATTCGGGGCCCGAGTAGCCAGAGTCCTGCAGCTCACTCGAAACCGCCACTCACCGTGGCTAATTGCCCATCAATAAAGGGCCCGGGCAGTGAGGAATTCCTCCGAAAGTCGGGTCCTCCGTTCTCCAGCCGAAGATTTTTTCGAGCAACCAAAATATTATGGTGTGCCCCGCTGTTCTCGCACAGTCAGCGCGAATTTGCTGCGGTGAGTCGATGCTGTTTCGCAGGACCTTCTTCCATTTTCGTCTCCCTCTTGCTCAGCCTGTCCCTGTTCCTCTGCAGTTCCCTATCTCCTGATGCCTGTGCTCCTTTGGCGGCACTGTGTCCTGTCGTCGTTGTTTTCCTGTGATTTGACATGTCTGTTAGCAGGATGCCTGACCCTGAGGCCGAGCCCTGGTCTCAGTGTCCACTGTTCCACTTTGATGTGATTCGTCAGTGCGGTGGACTACTGCTACTGCTCTCTTGCTGGACTGCGTCTTGAGTCCTGTTCGGCTGCCCCCTCCCGTGACCTCTGACCCTGCACTCTGCGGCTTTCCAGCGGCGTTTGTTGGCGAATCTGACCCCGAGCTCCTGCTGCTCCTTCGCTCCTTCGCTCCTTCTCCGCATCTCCGCTCTTTGGACTTCGTACGAATCAAAATTGGTCACAGCACCGAGTGAATTGCCCCGGAGACCGCAATGCGCTGTATTTATAGTAAACGTGTCCGATTGATTTGGCCACCCGTGGCGGCTCTGTCACAGATGCCTCAATTTGCATCTATCGAATGGTTTACATGGCTCTAAAAAGGTACC

24B

GTGGCAAAGTCAATCCGAATCACTTCAGTCTAATCAAATTAGACGGAGTGTAAGGAGATACATCAAAATCAAATCTGCTGGCTTGACAAAATGTTAATTTAATAAAACATTGCGTAGTACAACGTGAATTTAGGACAAGATAAATAGAATTGAAAATCAGCTAATCCAATTAAAGGCATTTGAGTGCGATTAATAAAATTAATTTTTTTTCAATAATCAATCTGTAGTAACCATTAATAATACTTGCATTCGAGACACTCTTGCTAATTTAATTCTTGAAAGAGAAAATTCTACTTAGTCTGAAGTAAATAATATATAAATTTCTTCTTTTTTTTAGGCGTCTCTCCATTCCAGAGCCTGTGCTCATGCTGCTCCCTGAAGCCCCCGCCGCCACCGGGCTCATCGGTGGTGGCTCCACTGAGCATTCCGGTCAGCAGCAGCTCGGCGGCCAGTTCGCCGGAATCGCCAAAGTCCTCCGGCCAGGGATCCGTTCATGATCCGCGGTCCAATTCGGTGGCCGAACTGCGGCGGAAGGCGCAGGAGCATTCGGCGGCACTTTTGCAATCGCTGCACGCGGCAGCTGCTGCAGGCTTGGCTTTTCCGGGCCTCCACTTGCCACCCCTGTCCTTCGCCCACCATCCGGCCTTGGGGCAGCATGTGGTCAATCACAACAATAACAACACGATGAGAATGAAGCACGAGGCTCAGGACATGACCATGAATGGTCTGGGCCCCGGCTCAGGATCTGGTTCGGGATCAGGTTCGGCAGGAGGCGGCACATCATCGGCCGCTCTTTTGGATCTGGCCGAATCGGCAGTTGCATATCAGCAGCAGCAACATGCCACATTGTCGCCACCAACCACGCCCACGCAGCAATCATCCGGGGGCGTGGCAGCAACGGAGGGCTCTCCAGGATCAGGAGCGATTGCAGGATCGGGTTCATTGAATGGAAACGTGGTGCTGACCAAAATGGAATAAACAATAGTTGGCCGAGGAAAAAACGAACAGAGTGTGCAATGAAAAGGCTGGGTATTTTATTTTTCAGATTTATAAATTTGTACAAACTTCAGGCCATCTTATTTCCACGCCATTTTCTTTGGTCTCCAAACAAAGTCCGAGAAAATCTTTAAAATGAAATACCCAAACCAGCGCGTTTGTAACAATAAACAATGAAAACATTTTAAAGTTTAATAG

**PolyA sites used**

*α-tubulin* polyadenylation signal

GCGTCACGCCACTTCAACGCTCGATGGGAGCGTCATTGGTGGGCGGGGTAACCGTCGAAATCAGTGTTTACGCTTCCAATCGCAACAAAAAATTCACTGCAACACTGAAAAGCATACGAAAACGATGAAGATTGTACGAGAAACCATAAAGTATTTTATCCACAAAGACACGTATAGCAGAAAAGCCAAGTTAACTCGGCGATAAGTTGTGTACACAAGAATAAAATCGGCCAGATTCAGTGTTGTCAGAAATAAGAAAACCCCACTGTTTTTCTTT

*α-tubulin* and two *SV40* polyadenylation signals

GCGTCACGCCACTTCAACGCTCGATGGGAGCGTCATTGGTGGGCGGGGTAACCGTCGAAATCAGTGTTTACGCTTCCAATCGCAACAAAAAATTCACTGCAACACTGAAAAGCATACGAAAACGATGAAGATTGTACGAGAAACCATAAAGTATTTTATCCACAAAGACACGTATAGCAGAAAAGCCAAGTTAACTCGGCGATAAGTTGTGTACACAAGAATAAAATCGGCCAGATTCAGTGTTGTCAGAAATAAGAAAACCCCACTGTTTTTCTTTGATATCAACTTGTTTATTGCAGCTTATAATGGTTACAAATAAAGCAATAGCATCACAAATTTCACAAATAAAGCATTTTTTTCACTGCATTCTAGTTGTGGTTTGTCCAAACTCATCAATGTATCTTAAACTTGTTTATTGCAGCTTATAATGGTTACAAATAAAGCAATAGCATCACAAATTTCACAAATAAAGCATTTTTTTCACTGCATTCTAGTTGTGGTTTGTCCAAACTCATCAATGTATCTTA

**Primer sets used for RT-qPCR quantification**

*RP49* (normalization control)

AAGCCCAAGGGTATCGACAACAGA

TGCACCAGGAACTTCTTGAATCCG

λ DNA

GTATTGGCCTCAGCATGGA

GGCGTTTCATTCCCGTTTG

PRE

GCCCCATAATTGTGACTTTGGGTC

AAGAGGAGTGGGGAGTCGGGGTG

RP2

CCTCCAATAAAGCGAATCCAGCGTGAG

CCAATCCGTTTAATCAGGCG

1

GTTGCCCGTTTTGATGCG

CCGACTTGTTGACTTTGCG

4+6

TGGGCTCTGGGCTCTGGACTATC

TCCTGCGGCTTTACGACGGTC

EL

GGCAGATGGGAACTGATGAA

ATCACACTTACTCCCTCGAAATC

3+7

GAAGGACATGCGTGAGTTTATTG

GCAGTTCCTAGTCCTACCTAGT

late

CGCAGGACCTTCTTCCATTTTCG

CAGGACACAGTGCCGCCAAAG

wD5

CTCCCCCTTTCTCACTCCC

CTTTCTACTCCGCTACACAGAA

24B

CAGCAACATGCCACATTGTC

ATGAACCCGATCCTGCAATC

*lacZ*

GCTGTGCCGAAATGGTCCATCAAA

TACTGACGAAACGCCTGCCAGTAT

*eve* (normalization control for *lacZ* in S3 Fig)

TCCAGTCCGGATAACTCCTTGAAC

TGTAGAACTCCTTCTCCAAGCGAC

**Specific primers used for producing cDNA**

(S3 Fig)

For EL specific primer: CCAGCAGATTCGGATACAGTAA

For late specific primer: TCCTGCCGAGCGAATCCAAC

For RP49 specific primer: GTGTATTCCGACCACGTTACA

# Reference

1. Thurmond J, Goodman JL, Strelets VB, Attrill H, Gramates LS, Marygold SJ, et al. FlyBase 2.0: the next generation. Nucleic Acids Res. 2019;47(D1):D759-D65.
